# Early Prediction of Heart Failure From Routine Cardiac CT Using Radiomic Phenotyping of Epicardial Fat

**DOI:** 10.1016/j.jacc.2026.02.5116

**Published:** 2026-06-30

**Authors:** Evangelos K. Oikonomou, Kenneth Chan, Parijat Patel, Elizabeth Wahome, Katerina Dangas, Rohan Desai, Ikboljon Sobirov, Rohan Khera, Steffen E. Petersen, Francesca Pugliese, Ronak Rajani, Edward Nicol, Attila Kardos, David Adlam, Andrew D. Kelion, Nikant Sabharwal, Nicholas Screaton, John P. Greenwood, Jonathan Rodrigues, Daniel Huck, Cheerag Shirodaria, Pete Tomlins, Muhammad Siddique, Yogesh Sohan, Sam Fry, Marly van Assen, Ron Blankstein, Milind Y. Desai, Stefan Neubauer, Keith M. Channon, John Deanfield, Charalambos Antoniades, ORFAN Consortium, Sheena Thomas, Jon Denton, Robyn Farrall, Wendy Qin, Mary Kasongo, Chrisha Ledesma, Damaris Darby, Ahmad Abdullrahman, Bruno Silva Santos, Alexios S. Antonopoulos, Christos P. Kotanidis, Susan Anthony, Adrian Banning, Cheng Xie, Rafail A. Kotronias, Lucy Kingham, Rajesh K. Kharbanda, Chris Mathers, Edward Nicol, Tarun K. Mittal, Jonathan, Weir-Macall, Attila Kardos, Anne Rose, David Adlam, George Hudson, Amrita Bajaj, Intrajeet Das, Aparna Deshpande, Praveen Rao, Dan Lawday, Francesca Pugliese, Steffen E. Petersen, Saeed Mirsadraee, Nicholas Screaton, Jonathan Rodrigues, David Murphy, Benjamin Hudson, John Graby, Colin Berry, Mohamed Marwan, Pál Maurovich-Horvat, Guo-Wei He, Wen-Hua Lin, Naohiko Takahashi

**Affiliations:** a Section of Cardiovascular Medicine, Department of Internal Medicine, Yale School of Medicine, New Haven, Connecticut, USA; b Division of Cardiovascular Medicine, Radcliffe Department of Medicine, University of Oxford, Oxford, United Kingdom; c Barts Heart Centre, St Bartholomew’s Hospital, Barts Health NHS Trust, London, United Kingdom; d William Harvey Research Institute, Barts and The London School of Medicine and Dentistry, Queen Mary University of London, London, United Kingdom; e Department of Cardiology, Guys and St Thomas’ Hospital, London, United Kingdom; f Royal Brompton and Harefield Hospitals, London, United Kingdom; g School of Biomedical Engineering and Imaging Sciences, King’s College, London, United Kingdom; h Department of Cardiology, Translational Cardiovascular Research Group, Milton Keynes University Hospital NHS Foundation Trust, Milton Keynes, United Kingdom; i Department of Cardiovascular Sciences, University of Leicester and NIHR Leicester Biomedical Research Centre, Leicester, United Kingdom; j Oxford University Hospitals NHS Foundation Trust, John Radcliffe Hospital, Oxford, United Kingdom; k Royal Papworth Hospital, Cambridge, United Kingdom; l Baker Heart and Diabetes Institute, Melbourne, Victoria, Australia; m Leeds Teaching Hospitals NHS Trust, Leeds, United Kingdom; n Royal United Hospital Bath NHS Foundation Trust, Bath, United Kingdom; o Division of Cardiovascular Medicine, Department of Medicine, Brigham and Women’s Hospital, Harvard Medical School, Boston, Massachusetts, USA; p Caristo Diagnostics, Oxford, United Kingdom; q Department of Radiology and Imaging Sciences, Emory University Hospital, Atlanta, Georgia, USA; r Cleveland Clinic Heart and Vascular Institute, Cleveland, Ohio, USA; s Institute of Cardiovascular Science, University College London, London, United Kingdom

**Keywords:** artificial intelligence, cardiac computed tomography, epicardial adipose tissue, heart failure, obesity, radiomics

## Abstract

**Background:**

Epicardial adipose tissue (EAT) is a metabolically active visceral fat depot that is both a sensor and a modulator of myocardial biology and changes its composition in response to paracrine signals from the myocardium. We hypothesized that radiomic characterization of EAT from routine coronary computed tomographic angiography (CCTA) can noninvasively capture this adverse remodeling and enable early heart failure (HF) risk stratification.

**Objectives:**

We sought to develop and externally validate a reproducible radiomic signature of EAT associated with incident HF.

**Methods:**

We conducted a multicenter cohort study of 72,751 adults without known HF or myocardial infarction undergoing CCTA across 9 UK centers (2007-2022). We deployed a fully automated pipeline to segment EAT and extract 1,655 volumetric, shape, and higher-order radiomic texture features. Using a harmonized survival autoencoder architecture, we derived the fat radiomic profile for HF (FRP_HF_). The model was developed in 59,327 individuals from 7 centers (age 57 ± 13 years, 47.5% female) and externally tested in 13,424 participants from 2 geographically distinct centers (58 ± 12 years, 49.4% female). Survival models were adjusted for age, sex, and conventional risk factors, including coronary artery disease (CAD) severity and EAT volume.

**Results:**

Over a median follow-up of 5.1 and 4.0 years, 1,737 (2.9%) and 363 (2.7%) participants developed HF in the internal and external validation cohorts, respectively. FRP_HF_ demonstrated robust discrimination (C-statistics: 0.869 [95% CI: 0.850-0.889] internal; 0.850 [95% CI: 0.831-0.870] external). Each 25-percentile increase in FRP_HF_ was associated with a nearly 4-fold higher adjusted HF risk (adjusted HRs: 3.90 [95% CI: 3.13-4.84] internal; 3.79 [95% CI: 3.01-4.76] external; both *P* < 0.001), with individuals in the highest decile exhibiting a nearly 20-fold higher risk compared with the lowest decile. In the external cohort, addition of FRP_HF_ to conventional risk models, including EAT volume and CAD severity, significantly improved 5-year discrimination (ΔAUC: 0.047; 95% CI: 0.029-0.065) and net reclassification (NRI: 0.39; 95% CI: 0.29-0.48) and suggested net clinical benefit on decision curve analysis. The associations were consistent across demographic subgroups and across the ejection fraction spectrum.

**Conclusions:**

Automated radiomic phenotyping of EAT from routine CCTA enables scalable, biologically informed stratification of future HF risk before clinical onset, positioning opportunistic imaging-based visceral fat profiling as a potential tool for precision prevention.

Obesity represents a major modifiable risk factor for heart failure (HF) and broader cardiovascular risk,^1^ with novel antiobesity medical therapies, such as incretin analogues, effectively reducing major adverse cardiovascular events even in the absence of diabetes mellitus.^2^ Initially, adipose tissue (AT) was recognized for its role in secreting proinflammatory adipokines and cytokines with deleterious endocrine and paracrine effects on the cardiovascular system.^3^ More recent research has highlighted substantial heterogeneity across AT depots, with visceral fat contributing disproportionately to the adverse cardiometabolic effects of obesity.^4^^,^^5^ Moreover, the metabolic profile of AT is increasingly recognized as a key modifiable factor and therapeutic target in obesity,^6^ as further supported by emerging evidence dissociating the cardiovascular benefit of incretin analogues from the observed weight loss.^2^^,^^7^

Epicardial adipose tissue (EAT) is a biologically active visceral fat depot that directly surrounds the human myocardium.^3^^,^^8^ Notably, EAT not only exerts paracrine effects on the human heart,^9^ but it can also sense inflammatory signals originating from the adjacent myocardium.^10^ Such inside-to-outside signaling induces changes in the adipocyte metabolic profile, resulting in the compensatory up-regulation of antiinflammatory adipokines, a phenomenon that is most pronounced in disease states, such as HF.^10^ During this process, EAT undergoes lipolysis, leading to a compositional shift from lipid-rich to aqueous-rich content in layers adjacent to the inflamed myocardium. These regions also exhibit marked fibrosis and angiogenesis, driven by profibrotic and proangiogenic signals originating from the myocardium.^11^ Thus, the ability of EAT to modify its texture and composition in response to early disease signals originating from the heart provides a direct window into myocardial biology. However, its clinical translation is limited by the lack of established imaging biomarkers that can characterize this phenotypic transition beyond simple volumetric expansion.

Coronary computed tomographic angiography (CCTA) has evolved into a first-line test for the investigation of coronary artery disease (CAD).^12^^,^^13^ Advances in artificial intelligence (AI) enable automated analysis of these scans to identify cardiovascular risk features that are not apparent on standard clinical interpretation.^11^^,^^14^, ^15^, ^16^ This paradigm previously led to the development of the pericoronary fat radiomic profile (FRP),^15^ which has demonstrated reproducible associations with coronary inflammation, a process that inhibits perivascular adipogenesis and promotes lipolysis, fibrosis, and angiogenesis in perivascular fat, with prognostic implications for major adverse cardiac events.^11^^,^^14^, ^15^, ^16^ Nevertheless, efforts to define reproducible radiomic biomarkers linking global epicardial adiposity to the risk of HF have been limited by the lack of large multicenter imaging studies, a paucity of algorithmic approaches to handle technical confounders and noise,^17^ and a technical inability to capture the 3-dimensional textural changes of EAT in response to the myocardial paracrine signals.^15^^,^^18^

We hypothesized that noninvasive characterization of the EAT texture and composition may flag this early remodeling, thus enabling accurate stratification of future HF risk. We tested this hypothesis with the use of a deep learning–enabled radiomic approach that characterizes epicardial adiposity in routine CCTA scans collected as part of the ORFAN (Oxford Risk Factors and Noninvasive Imaging; NCT05169333) study, the largest multicenter cohort linking CCTA data with centralized electronic health records (EHR). The overall objective was to define a scalable technology for the screening of patients undergoing CCTA as part of clinical care, enabling detection of early signals of HF, that may inform timely interventions to improve clinical outcomes.^2^^,^^19^, ^20^, ^21^

## Methods

### Study design and participants

The ORFAN study was approved by the Oxfordshire Research Ethics Committee (REC 15/SC/0,545). At the time of the analysis, the ORFAN study had enrolled a sample of 87,987 studies from 81,187 unique patients undergoing CCTA as part of routine clinical care across 9 different hospitals and trusts across the UK from 2007 to 2022. Eligible patients were 18 years of age or older at the time of CCTA and had not opted out from use of their clinical data for research. From this initial sample, we restricted our analysis to the chronologically first study per participant and excluded individuals who had established HF (including previous history of ventricular assist device placement or heart transplantation) or a history of myocardial infarction (MI). To further account for the possibility of delayed entry of a diagnosis code in the chart, we also excluded individuals who had either event recorded within 30 days of the scan.

Patient demographics and clinical outcomes data were collected via local EHR and nationwide databases (NHS England) using International Classification of Diseases, 10th revision (ICD-10) codes. Detailed characteristics of HF diagnosis and ischemic heart disease (IHD) were collected from the National Institute of Cardiovascular Outcomes Research (NICOR) datasets, including National Heart Failure Audit, Myocardial Ischaemic National Audit Project, and National Audit of Percutaneous Coronary Interventions. Outcomes data were collected until March 31, 2023.

**Figure 1:**
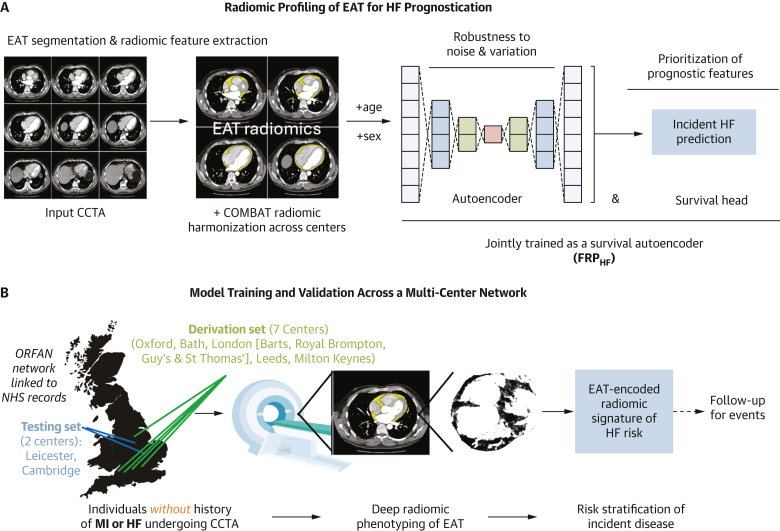
Radiomic Pipeline for EAT Profiling (A) Our end-to-end radiomic pipeline involves automated detection of the pericardial border, followed by extraction of the epicardial adipose tissue (EAT) and direct computation of 1,655 radiomic features. These, along with age and sex, are passed through a site-level harmonization process and then fed through a survival autoencoder. This architecture is designed to maximize the signal-to-noise ratio of radiomic signatures, while learning reproducible signatures linked to incident heart failure (HF). (B) The full algorithmic pipeline was trained in a development set of 7 centers (n = 59,327) and evaluated in an independent test set of 2 geographically distinct centers (n = 13,424) across the UK. The study population included individuals without known HF or myocardial infarction (MI) and with a 30-day blanking period. CCTA = coronary computed tomographic angiography; EAT = epicardial adipose tissue; FRP_HF_ = fat radiomic phenotype for heart failure; HF = heart failure; MI = myocardial infarction; NHS = National Health Service; ORFAN = Oxford Risk Factors and Noninvasive Imaging cohort.

The data were subsequently split at a center level into a development set (7 centers: Oxford University Hospitals, Royal United Hospital Bath, Royal Brompton, Barts Hospital, Milton Keynes Hospital, Leeds Teaching Hospitals, Guy’s and St Thomas’ Hospitals), and a geographically distinct test set (2 randomly selected centers: Royal Papworth Hospital and Leicester University Hospital) for unbiased external evaluation of model performance. The development set was further randomly split into a training and an internal validation cohort. A flowchart of inclusions and exclusions is presented in Supplemental Figure 1, and an overview of the study design is shown in Figure 1.

All patient information was collected under Section 251 of the UK National Health Service Act 2006, following approval by the UK Confidentiality Advisory Group (20/CAG/0,157). Eligible CCTA scans were transferred to the ORFAN study core laboratory at the Acute Multidisciplinary Imaging and Interventional Centre at the University of Oxford, using a General Data Protection Regulation–compliant gateway (CIMAR gateway, Caristo Diagnostics).

### Key definitions and outcomes

For the purposes of this study, key exposures and outcomes were defined based on local and nationwide databases and EHR and standard diagnosis (ie, ICD-10) and procedure (ie, Operating Procedure Codes Supplement 4) codes (Supplemental Table 1). Key risk factors (eg, hypertension, diabetes mellitus) were defined based on the date of first appearance in the national EHR database. The primary outcome was the incidence of new-onset HF, defined as the first appearance of an HF-specific ICD-10 code ≥30 days after the index CCTA scan among included at-risk individuals without known HF or previous MI. These clinical outcomes were cross-validated against the events recorded in the NICOR datasets, with further classification into HF with reduced (HFrEF), mildly reduced (HFmrEF), or preserved ejection fraction (HFpEF) based on the corresponding echocardiographic left ventricular ejection fraction value (≤40%, 41%-49%, and ≥50%, respectively), where available.^22^ To evaluate the specificity of the trained radiomic model for incident HF relative to other major adverse cardiovascular events, we also evaluated the association with all-cause mortality, cardiac mortality, and incident MI as key secondary outcomes (Supplemental Table 1). We further stratified HF events into those occurring in the presence or absence of IHD based on the concurrent presence or absence of a related diagnosis code.

### Series selection and automated segmentation of cardiac structures

The series selection within each study and subsequent segmentation of cardiac structures (pericardium, coronary centerlines, coronary wall, and perivascular adipose tissue [PVAT]) were performed using CaRi-Heart V2.5 (Caristo Diagnostics). The device uses deep learning models for the automated segmentation of these structures,^23^ which were further calibrated as described in the Supplemental Methods.

### EAT radiomic feature extraction

The full process for quality control, training, validation of the segmentation steps, and extraction of the radiomic features is described in the Supplemental Methods. Briefly, we first used a validated residual U-Net–based convolutional neural network that performs automated segmentation of the pericardial border from input CCTA scans provided in standard (DICOM) format, as previously described.^23^ In line with previous work, standard attenuation thresholds of −190 to −30 Hounsfield units (HU) were then applied to all voxels found within the region of interest defined by the pericardial segmentation to isolate EAT.^11^^,^^14^^,^^15^^,^^23^ Using this input mask, we computed a total of 1,655 radiomic features, spanning shape and first-order (eg, volumetric, shape, and histogram-derived metrics) and higher-order radiomic features reflecting complex spatial relationships in the distribution of attenuation values across the EAT depot, with the use of the RAD-Extractor of the CaRi-Heart toolkit. This approach includes additional wavelet transformations that capture multiscale textural patterns in EAT by decomposing the image into low- and high-frequency (L vs H) components across different spatial directions (eg, HHL, LLL). This was done to enhance the characterization of attenuation heterogeneity, allowing extraction of fine and coarse structural details across all 3 spatial dimensions. For each scan, we then compiled all radiomic features, along with age and recorded sex at the time of the scan, into a 1,657-dimensional array (1,655 radiomic features + age + sex) that was used for model training and subsequent evaluation. This ensured that the learned radiomic signature accounted for differences in age and sex when evaluating potential radiomic combinations linked to increased HF risk.

### Developing an EAT-specific signature of HF risk

In the training set, all radiomic features (along with age and sex) were first processed through a ComBat harmonization layer,^24^ which performs batch-level harmonization at the site level to address systematic variations introduced by different imaging protocols or scanner parameters across centers. This method relies on bayesian hierarchic modeling, which estimates and removes site-specific biases while preserving biological variability, ensuring that feature distributions across imaging centers are similar while minimizing confounding effects that could otherwise bias the learned representations. Harmonization parameters were estimated in the training set and applied to the validation and test sets to avoid information leakage. Processed features were then entered into a survival autoencoder.^25^ The first part consisted of an encoder-decoder architecture with hidden dimensions [32, 16, 8], incorporating ReLU activations, batch normalization, and dropout (*P* = 0.3) to enable compression of the 1,657 features. This was intended to regularize feature learning, extract meaningful representations from high-dimensional radiomic data, and reduce noise without losing discriminative power. The last part consisted of a survival prediction head, a single-layer linear Cox regression layer that operates on the latent space and produces a continuous log-risk score proportional to the hazard ratio. We trained with the use of a weighted loss function that combines the reconstruction mean squared error loss from the autoencoder and the Cox partial log-likelihood loss for survival modeling:

$$L=MSE+L_{Cox}$$

Model training was conducted using the Adam optimizer with a learning rate of 1 × 10^−3^, batch size of 64, and early stopping set at 3 consecutive epochs of nonimprovement in validation loss. The model was trained for up to 100 epochs, using an Nvidia Tesla V100 GPU with 32 GB VRAM. To prevent overfitting, we implemented an early stopping function if the validation loss failed to improve after 3 epochs. Finally, we transformed the output of the survival layer (log-risk score) predictions into percentiles based on the respective distribution in the development set (to avoid data leakage into the testing set) and facilitate interpretation of population-level comparisons.

### CT-derived covariates

To enable internally consistent adjustment for imaging-relevant phenotypes, we extracted several CT-derived covariates for inclusion in our multivariable models. CAD burden was quantified according to the CAD-RADS 2.0 classification derived from CCTA reports (categories 0 to 5, with a separate category when insufficient information from the original report precluded a reliable ascertainment).^26^ EAT volume was computed from the same automated pericardial segmentation used for radiomic feature extraction. When anthropometric parameters were missing, body mass index (BMI) was derived by estimating height and weight directly from CCTA images with the use of dedicated deep learning models (Supplemental Methods). Where possible, the 10-year PREVENT-HF risk was estimated with the data available at the time of the scan, using the previously validated formulas.^27^

### Model explainability

To provide explainable insights into the relative contribution of individual radiomic features, we first used Shapley additive explanations (SHAP) values.^28^ Briefly, SHA*P* values estimate the impact of each radiomic feature on model predictions by averaging its contribution across all possible feature combinations, providing a globally consistent measure of importance. We also retrieved representative examples of individuals with visually similar EAT distribution and volume, but in whom radiomic phenotyping revealed substantial differences in the FRP_HF_ profile linked to differences in the risk of incident HF.

### Evaluating the specificity of EAT vs pericoronary radiomic profiling

To better understand the specificity of EAT radiomic profiling through FRP_HF_ vs pericoronary fat-specific imaging, we recalibrated FRP_PVAT_, as previously described,^15^ for prediction of MI events in the ORFAN population. All automated segmentations of the coronaries and surrounding PVAT were performed within CaRi-Heart v2.5, by applying an unsupervised fully automated segmentation pipeline. From the PVAT masks we computed a total of 4,965 radiomic features (1,655 radiomic features computed around each one of the main 3 coronary vessels, namely, the right coronary artery, left anterior descending artery and left circumflex artery) (Supplemental Methods) which were processed through the same age- and sex-adjusted survival autoencoder architecture described above. This enabled a direct head-to-head comparison of global vs pericoronary EAT radiomic profiles in their specificity for predicting HF vs MI events.

### Statistical analysis

Continuous variables are summarized as mean ± SD or median (Q1-Q3) and categoric variables as n (%). Univariate correlations between continuous variables were examined by means of linear regression models or nonparametric Spearman rho, as appropriate. We evaluated the association between the FRP_HF_ percentiles and incident HF in unadjusted Nelson-Aalen cumulative hazard curves presented across representative strata of radiomic profile percentiles, as well as in multivariable Cox regression models with FRP_HF_ percentiles, along with age, sex, traditional cardiovascular risk factors (EAT volume, BMI, hypertension, diabetes mellitus, chronic kidney disease, history of IHD, MI, peripheral arterial disease, and stroke) and CAD-RADS. Using similar multivariable models, we estimated the cumulative incidence of HF at 1, 3, and 5 years across EAT radiomic percentile scores, which was graphically presented for a restricted cubic spline Cox model (with k = 3 knots). We further evaluated model robustness by accounting for all-cause mortality as a competing risk and by incorporating center-level clustering using Cox proportional hazards models with Huber-White sandwich estimators clustered at the site level.

Time-dependent discrimination metrics were estimated at prespecified intervals (5 years) and were used to plot time-dependent AUCs for a series of models. Model 1 included age, sex, and EAT volume; model 2 further incorporated all the above-mentioned clinical risk factors (establishing the baseline reference); model 3 included only age, sex, and FRP_HF_ alone; and model 4 incorporated FRP_HF_ percentile scores into the baseline model (model 2). The difference in discrimination between the baseline and FRP_HF_-enhanced models (model 2 vs 4) was evaluated with the use of DeLong’s test for paired comparisons against the baseline model. Net reclassification improvement was examined using the continuous net reclassification index (NRI), whereas clinical utility was estimated with the use of decision curve analysis, estimating the net benefit of models incorporating FRP_HF_ across a range of clinically relevant risk thresholds. Where appropriate, 95% CIs were computed by means of bootstrapping with 500 replications. Calibration was evaluated by estimating predicted versus observed absolute risks at 5 years for key risk groups (<2.5%, 2.5%-9.9%, 10%-19.9%, ≥20%) using the Greenwood-d’Agostino-Nam test and statistic.^29^ These were selected as clinically interpretable strata commonly used in previous HF risk prediction and prevention frameworks, driving clinical decision making.^27^

In subgroup analyses, to ensure model convergence across subgroup analyses due to the reduced event counts, any subgroup estimates (across age, sex, and race groups) were adjusted only for age and sex. In further analyses, we evaluated the cross-sectional association between FRP_HF_ and each one of BMI, EAT volume, and 10-year PREVENT-HF risk, reporting the proportion of variance explained in cross-sectional linear regression analyses. Finally, we examined the correlation between FRP_HF_ with FRP_PVAT_ with the use of linear regression, as well as the adjusted association of each one of these biomarkers with a range of outcomes in separate multivariable Cox regression models.

We set alpha (α) at 0.05 with no adjustment for multiple comparisons. Statistical analyses and visualizations were performed using Python version 3.11 (Python Software Foundation) and R version 4.2.3 (R Foundation). Reporting stands consistent with the TRIPOD-AI^30^ statement and PRIME 2.0 guidelines.^31^

## Results

### Study population

From 81,187 unique individuals in the ORFAN cohort, 7,389 (9.1%) had existing HF or known history of baseline or previous MI events at the time of undergoing CCTA, 1,015 (1.5%) had a new diagnosis of HF or MI or died less than 30 days after CCTA, and 32 (0.04%) had incomplete radiomic feature extraction. After excluding these patients, our study sample consisted of 72,751 individuals without HF or previous MI who underwent CCTA and had at least 30 days or more of follow-up across 9 UK hospitals. The study population flowchart is shown in Supplemental Figure 1, and study population demographic characteristics are summarized in Table 1. Eligible participants were split at the center level into a development set (7 centers, used for training and internal validation) and a geographically distinct external validation set (2 centers) (Figure 1, Supplemental Figure 1). The former included 59,327 unique participants (age 56.6 ± 13.2 years, 28,153 [47.5%] women), with 1,737 (2.9%) new HF events (≥30 days) reported over a median follow-up of 5.1 years (Q1-Q3: 3.4-7.6 years). Among these, 47,461 individuals (80.0%) were randomly sampled in the training set, and the remaining 11,866 (20.0%) were included in the internal validation set. The external validation set included 13,424 unique participants from 2 geographically distinct centers (age 57.8 ± 12.2 years, 6,633 [49.4%] women), with a median follow-up of 4.0 years (Q1-Q3: 3.0-6.9 years) and 363 (2.7%) new HF events.

**Table 1:** Baseline Characteristics

	Training (n = 47,461)	Internal Validation (n = 11,866)	External Validation (n = 13,424)
Age, y	56.6 ± 13.2	56.6 ± 13.2	57.8 ± 12.2
Sex			
Female	22,490 (47.4)	5,663 (47.7)	6,633 (49.4)
Male	24,971 (52.6)	6,203 (52.3)	6,791 (50.6)
Ethnicity			
Asian or Asian British	5,912 (12.5)	1,429 (12.0)	1,601 (11.9)
Black or Black British	2,672 (5.6)	638 (5.4)	228 (1.7)
Mixed ethnic group	526 (1.1)	122 (1.0)	410 (3.1)
Other ethnic group	13,498 (28.4)	3,458 (29.1)	548 (4.1)
Unknown	1,686 (3.6)	421 (3.5)	599 (4.5)
White	23,167 (48.8)	5,798 (48.9)	10,038 (74.8)
Body mass index, kg/m^2^	29.1 (26.2-32.5)	29.1 (26.1-32.6)	29.5 (26.3-32.9)
Baseline risk factors (before CCTA)			
Diabetes mellitus	5,901 (12.4)	1,405 (11.8)	1,371 (10.2)
Hypertension	15,352 (32.3)	3,830 (32.3)	4,234 (31.5)
Ischemic heart disease	7,233 (15.2)	1,768 (14.9)	2,326 (17.3)
Peripheral arterial disease	218 (0.5)	68 (0.6)	58 (0.4)
Stroke	1,216 (2.6)	294 (2.5)	333 (2.5)
Chronic kidney disease	1,492 (3.1)	372 (3.1)	275 (2.0)
Baseline medications (before CCTA)			
ACE inhibitors	5,077 (10.7)	1,234 (10.4)	1,774 (13.2)
ARBs	2,716 (5.7)	646 (5.4)	964 (7.2)
Beta-blockers	8,405 (17.7)	2,058 (17.3)	2,520 (18.8)
SGLT2I	492 (1.0)	97 (0.8)	160 (1.2)
MRA	566 (1.2)	138 (1.2)	157 (1.2)
Diuretics	2,427 (5.1)	591 (5.0)	858 (6.4)
Antiplatelets	5,117 (10.8)	1,227 (10.3)	2,192 (16.3)
Calcium-channel blockers	5,660 (11.9)	1,409 (11.9)	1,897 (14.1)
Statins	9,818 (20.7)	2,410 (20.3)	3,368 (25.1)
Ezetimibe	332 (0.7)	69 (0.6)	139 (1.0)
Incretin analogues	218 (0.5)	45 (0.4)	59 (0.4)
Valvular heart disease			
Baseline aortic valve disease	5,544 (11.7)	1,401 (11.8)	1,305 (9.7)
Baseline mitral valve disease	5,039 (10.6)	1,256 (10.6)	1,367 (10.2)
Baseline aortic/mitral valve disease	7,865 (16.6)	1,965 (16.6)	1,977 (14.7)
Technical parameters			
Scanner type			
Canon Medical Systems Aquilion	70 (0.1)	21 (0.2)	0 (0.0)
GE Medical Systems Discovery	6 (0.0)	1 (0.0)	2 (0.0)
GE Medical Systems Lightspeed	1,356 (2.9)	350 (2.9)	2 (0.0)
GE Medical Systems Revolution	0 (0.0)	0 (0.0)	6 (0.0)
GE Medical Systems Optima	4,566 (9.6)	1,115 (9.4)	1 (0.0)
Philips Brilliance	2 (0.0)	1 (0.0)	5 (0.0)
Philips Ingenuity	1 (0.0)	0 (0.0)	1 (0.0)
Philips iCT	1,954 (4.1)	498 (4.2)	0 (0.0)
Siemens Biograph	2 (0.0)	0 (0.0)	3 (0.0)
Siemens Somatom Definition	12,672 (26.7)	3,277 (27.6)	7,663 (57.1)
Siemens Somatom Drive	22,150 (46.7)	5,467 (46.1)	5,336 (39.7)
Siemens Sensation	567 (1.2)	151 (1.3)	27 (0.2)
Toshiba Aquilion	4,112 (8.7)	985 (8.3)	374 (2.8)
Not specified	3 (0.0)	0 (0.0)	4 (0.0)
Tube voltage, kVp			
70	2,286 (4.8)	562 (4.7)	657 (4.9)
80	9,042 (19.1)	2,255 (19.0)	1,507 (11.2)
90	4,020 (8.5)	966 (8.1)	1,167 (8.7)
100	16,621 (35.0)	4,151 (35.0)	5,437 (40.5)
110	856 (1.8)	201 (1.7)	136 (1.0)
120	13,225 (27.9)	3,385 (28.5)	4,277 (31.9)
130	193 (0.4)	45 (0.4)	10 (0.1)
135	32 (0.1)	1 (0.0)	16 (0.1)
140	1,182 (2.5)	300 (2.5)	214 (1.6)
150	1 (0.0)	0 (0.0)	0 (0.0)
CAD-RADS classification^a^			
0	17,811 (37.5)	4,480 (37.8)	3,740 (27.9)
1	11,031 (23.2)	2,727 (23.0)	1,661 (12.4)
2	3,923 (8.3)	984 (8.3)	659 (4.9)
3	1,013 (2.1)	259 (2.2)	178 (1.3)
4	4,074 (8.6)	1,011 (8.5)	864 (6.4)
5	1,158 (2.4)	273 (2.3)	298 (2.2)
Outcomes (≥30 days after CCTA)			
Follow-up, y	5.1 (3.4-7.6)	5.1 (3.4-7.7)	4.0 (3.0-6.9)
Heart failure events	1,420 (3.0)	317 (2.7)	363 (2.7)
Myocardial infarction events	1,424 (3.0)	349 (2.9)	428 (3.2)
Death (all-cause)	4,311 (9.1)	1,103 (9.3)	1,090 (8.1)
Cardiac death	1,087 (2.3)	282 (2.4)	297 (2.2)

Values are n (%), mean ± SD, or median (Q1-Q3).

ACE = angiotensin-converting-enzyme; ARB = angiotensin receptor blocker; CAD-RADS = coronary artery disease–reporting and data system; CCTA = coronary computed tomographic angiography; MRA = mineralocorticoid receptor antagonist; SGLT2I = sodium-glucose cotransporter 2 inhibitor.

a Reported for those with available reports.

### EAT radiomic profiling and incident HF

**Figure 2:**
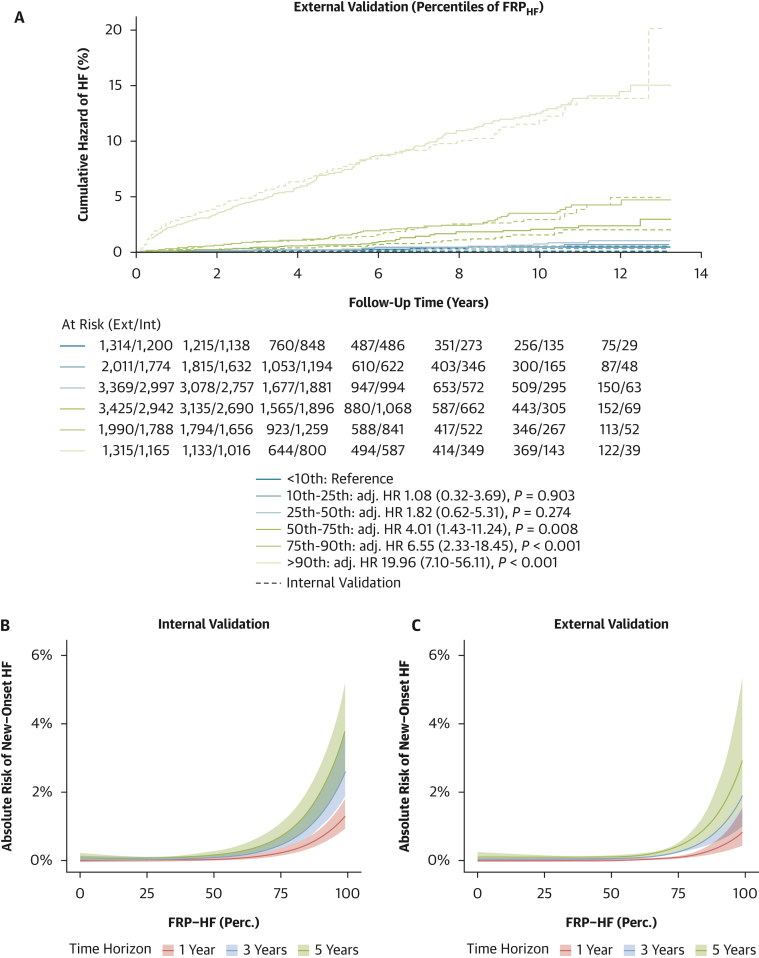
EAT Radiomic Profiling and Risk of New-Onset HF (A) Nelson-Aalen cumulative hazard curves for incident HF across discrete percentile groups (<10th, 10-25th, 25-50th, 50-75th, 75-90th, and >90th) of FRP_HF_ score in the internal (dashed lines) vs external validation sets (solid lines). The curves present the observed (unadjusted) cumulative hazard and are accompanied by HRs (and 95% CIs) derived from multivariable Cox regression models adjusted for age, sex, and traditional cardiovascular risk factors, including epicardial adipose tissue volume, body mass index, and CAD-RADS categories. (B, C) Estimated cumulative incidence of new HF diagnosis at 1 (red), 3 (blue) and 5 (green) years after CCTA across EAT radiomic score percentiles (x-axis) in the internal and external validation cohorts (modeled for the median of each covariate and assuming no categoric risk factors). The shaded areas denote the 95% CIs. CAD-RADS = coronary artery disease–reporting and data system; other abbreviations as in Figure 1.

Using the custom survival autoencoder architecture, we trained a global EAT radiomic signature (FRP_HF_) to predict incident HF events among 47,461 unique individuals in the training cohort. The final model showed robust discrimination for new HF events ≥30 days after CCTA, with a C-index of 0.883 (95% CI: 0.875-0.893) in the training set, 0.869 (95% CI: 0.850-0.889) in the internal validation set, and 0.850 (95% CI: 0.831-0.870) in the external validation set. After adjusting for age, sex, conventional risk factors (BMI, EAT volume, hypertension, diabetes mellitus, chronic kidney disease, history of IHD, peripheral arterial disease, and stroke) and CAD-RADS, we observed that for every quartile (25-percentile) increase in FRP_HF_, there was a 3.9-fold increase in HF incidence across the internal and external validation cohorts (internal adjusted HR: 3.90 [95% CI: 3.13-4.84]; external adjusted HR 3.79 [95% CI: 3.01-4.76]; both *P* < 0.001). Supplemental Table 2 presents unadjusted and various adjusted estimates. There was a dose-response association, with the absolute risk of new-onset HF increasing substantially above the 50th percentile of FRP_HF_, and those in the >90th percentile exhibiting a nearly 20-fold (adjusted HR: 19.96; 95% CI: 7.10-56.11; *P* < 0.001) higher risk of HF compared with those in the ≤10th percentile (Figure 2).

### FRP_HF_ vs traditional cardiovascular risk factors

**Figure 3:**
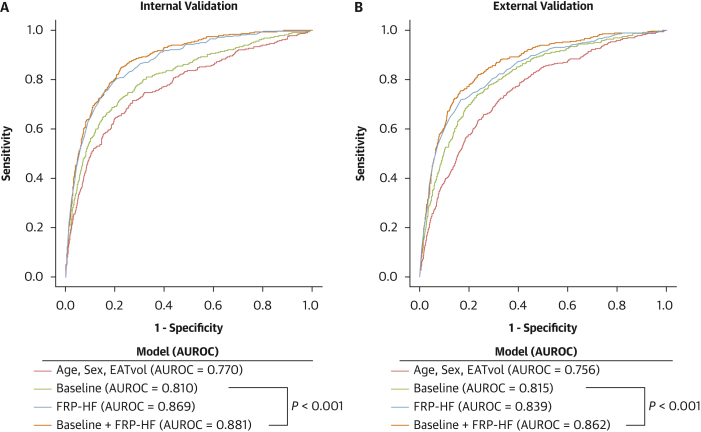
Discrimination of New-Onset HF at 5 Years According to FRP_HF_ vs Traditional Risk Factors Plots summarizing the area under the time-dependent receiver operating characteristic curve (AUROC) for discriminating incident HF cases at 5-years in the (A) internal vs (B) external validation sets. Four main different models are presented: 1) a model consisting of age, sex, and EAT volume alone (model 1, red line); 2) a baseline model consisting of age, sex, traditional cardiovascular risk factors (body mass index EAT volume, diabetes mellitus, hypertension, history of ischemic heart disease, myocardial infarction, stroke, peripheral arterial disease, chronic kidney disease) and CAD-RADS categories (model 2, green line), 3) an FRP_HF_-only model (model 3, blue line), and 4) a composite model incorporating FRP_HF_ alongside the baseline model predictors (model 4, orange line). *P* values represent the significance of pairwise comparisons against the baseline model (model 4 vs model 2). Abbreviations as in Figures 1 and 2.

Across both validation sets, the addition of FRP_HF_ to a baseline model consisting of age, sex, conventional risk factors, and CAD-RADS, significantly improved discrimination of new-onset HF (internal: 5-year AUC: 0.881 vs 0.810; ΔAUC: 0.071 [95% CI: 0.051-0.092; *P* < 0.001]; external: 5-year AUC 0.862 vs 0.815; ΔAUC: 0.047 [95% CI: 0.029-0.065; *P* < 0.001) (Figure 3). Adding FRP_HF_ to baseline risk factors also improved risk classification (continuous NRI: 0.39; 95% CI: 0.29-0.48; *P* < 0.001) (Supplemental Figure 2), whereas decision curve analysis demonstrated higher net benefit for models incorporating FRP_HF_ across clinically relevant 5-year HF risk thresholds from 5% to 15% (Supplemental Figure 3). There was good calibration for HF-free survival between the observed and predicted risk at 5 years (Greenwood-d’Agostino-Nam *P* value = 0.055) (Supplemental Figure 4).

**Figure 4:**
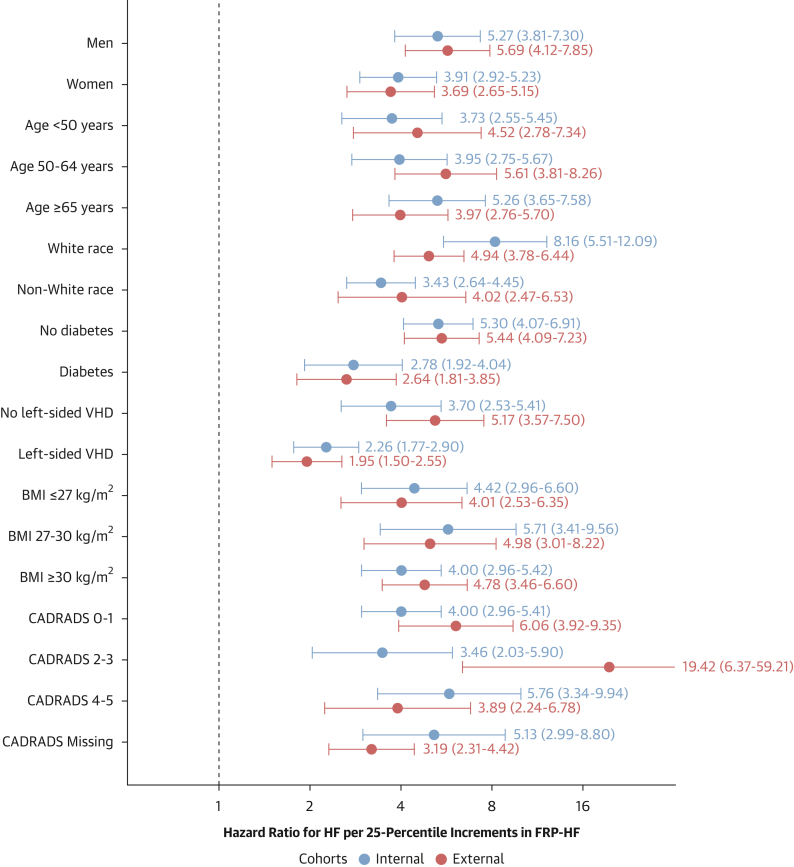
Association of FRP_HF_ With Incident HF Across Clinical Subgroups Forest plot presenting the age- and sex-adjusted hazard estimates for the association between FRP_HF_ (in quartile [25-percentile] increments) and incident HF across the internal (blue) and external (red) validation sets in relevant clinical subgroups defined at the time of the CCTA scan. BMI = body mass index; VHD = valvular heart disease (includes aortic stenosis/regurgitation and/or mitral stenosis/regurgitation; severity unspecified); other abbreviations as in Figure 1.

In key age- and sex-adjusted subgroup analyses, the model showed consistent performance across subgroups, including male and female, older and younger, different racial/ethnic backgrounds, across the range of CAD-RADS classes, and regardless of the presence of diabetes mellitus or known left-side valvular heart disease (Figure 4). Notably, in sensitivity analyses with linkage to echocardiographic parameters, the association was consistent across the ejection fraction spectrum, with higher FRP_HF_ linked to higher incidence of both HFrEF/HFmrEF and HFpEF events (adjusted HRs: 2.81 [95% CI: 1.04-7.55] vs 3.45 [95% CI: 1.24-9.55] per 25-percentile increments, respectively) (Supplemental Figure 5).

### FRP_HF_ vs traditional adiposity, inflammation, and HF biomarkers

To better understand the importance of EAT radiomic profiling beyond simple or traditional noninvasive imaging biomarkers of human adiposity, we further evaluated the interplay between BMI, CT-defined EAT volume and attenuation, and FRP_HF_. In the external set, BMI explained just 1.4% of the variance in the FRP_HF_ (*R*^2^ = 0.014; 95% CI: 0.009-0.022), and EAT volume similarly explained just a fraction (*R*^2^ = 0.065; 95% CI: 0.058-0.072). For reference, PREVENT-HF explained 18% (*R*^2^ = 0.18; 95% CI: 0.14-0.22) of the variance in FRP_HF_ across the testing sets (Supplemental Figure 6). Interestingly, in 12,053 individuals from the external set without diabetes mellitus at baseline, greater FRP_HF_ was associated with incident diabetes mellitus (n = 789 new diagnoses) in unadjusted models (unadjusted HR per 25-percentile increment: 1.20; 95% CI: 1.02-1.41), but the association was attenuated after adjusting for EAT volume alongside age and sex (adjusted HR per 25-percentile increment: 1.08; 95% CI: 0.94-1.24).

Finally, in a pooled analysis of 5,151 and 509 patients, respectively, from the internal and external validation sets with high-sensitivity C-reactive protein (hsCRP) and N-terminal pro–B-type natriuretic peptide (NT-proBNP) measurements recorded within 1 year of CCTA (±365 days), we observed a significant, yet weak correlation between higher FRP_HF_ and higher circulating inflammatory (hsCRP: *R*^2^ = 0.043; *P* < 0.001) and NT-proBNP (*R*^2^ = 0.109; *P* < 0.001) levels (Supplemental Figure 7). Finally, in a sensitivity analysis, excluding patients with previously measured ejection fraction <50% before the CCTA scan or NT-proBNP levels ≥300 pg/mL resulted in a stronger nominal effect size in the association between FRP_HF_ and new-onset HF (n = 13,407 observations; adjusted HR per 25 percentile increment: 8.49; 95% CI: 2.30-31.34).

### FRP_HF_ vs FRP_PVAT_

**Figure 5:**
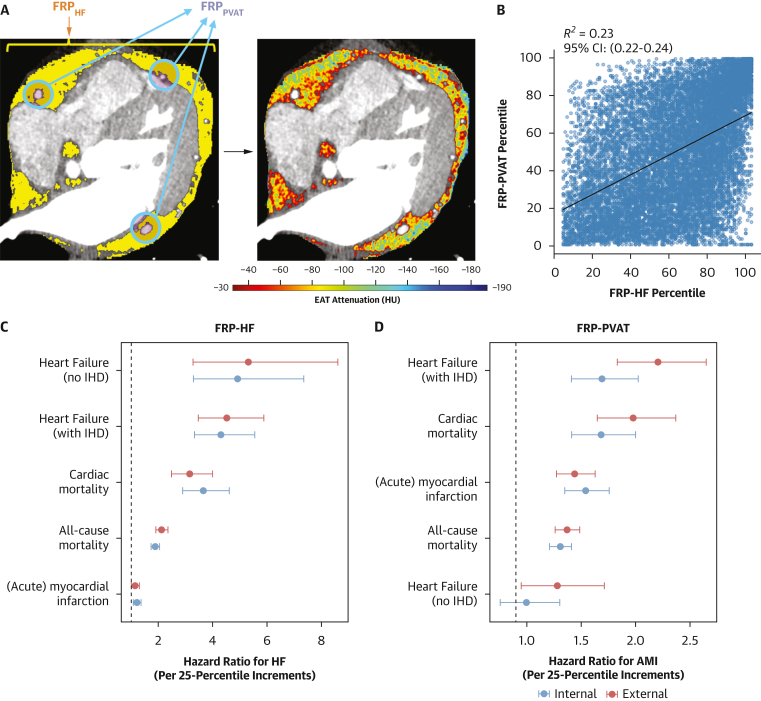
Global Epicardial vs Focal Perivascular Radiomic Profiling (A) Representative axial slice illustrating the segmentation of the EAT depot used in the development of the FRP_HF_ score, compared with a schematic visualization of the coronary perivascular adipose tissue (PVAT) surrounding the 3 main coronary arteries for development of the FRP_PVAT_ score. (B) Correlation of FRP_HF_ with FRP_PVAT_ in the external validation cohort. (C, D) Age- and sex-adjusted association of (C) FRP_HF_ and (D) FRP_PVAT_ with HF events in the presence or absence of ischemic heart disease (IHD), as well as all-cause and cardiac mortality and acute myocardial infarction (MI). HU = Hounsfield units; other abbreviations as in Figure 1.

To understand the value of radiomic profiling of distinct EAT regions, we evaluated the cross-sectional correlation between FRP_HF_ (the global EAT signature of HF risk) and FRP_PVAT_ (the regional coronary PVAT signature of MI risk, computed according to our previous work using 1,655 radiomic features for each one of the main 3 vessels)^15^ (Supplemental Methods, Figure 5A). Less than one-fourth (*R*^2^ = 0.23; 95% CI: 0.22-0.24) of the total variance seen in FRP_HF_ was explained by FRP_PVAT_, and vice versa (Figure 5B). Critically, global epicardial vs focal pericoronary radiomic profiling exhibited specificity for predicting future HF vs vascular events, respectively. For example, FRP_HF_ showed consistent performance for HF events regardless of the presence or absence of IHD (Figure 5C), whereas FRP_PVAT_ exhibited relative specificity for MI as well as HF events only in the presence of IHD (Figure 5D).

### FRP_HF_ enables explainable predictions

**Figure 6:**
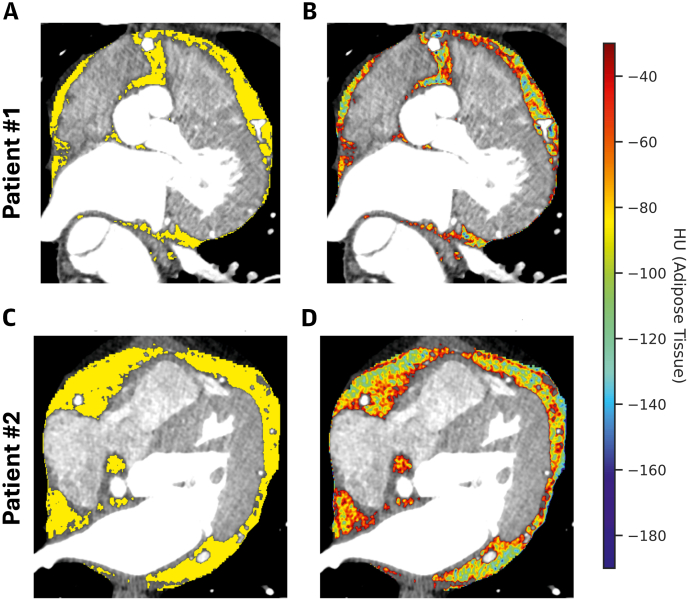
Example of Volumetric vs High-Throughput Radiomic Phenotyping of EAT (A) Example of EAT segmentation from an axial slice from a male patient, (B) with radiomic phenotyping by FRP_HF_ revealing an elevated risk of incident heart failure with an FRP_HF_ score in the 95th percentile. (C, D) Related example from a different case with radiomic EAT phenotyping revealing a more benign EAT remodeling profile with an FRP_HF_ in the 39th percentile. HU = Hounsfield units; other abbreviations as in Figure 1.

In explainability assessments based on SHAP (Shapley Additive Explanation values), we observed that shape (geometric) features, including least axis length, flatness, volume, and surface area of epicardial adiposity, and higher-order radiomic texture features reflecting the spatial distribution of attenuation patterns around the myocardium (eg, zone entropy, gray-level variance) ranked among the top contributors in terms of feature importance, underscoring the need for multiparametric characterization of epicardial adiposity for cardiac risk stratification (Supplemental Figure 8). In Figure 6, we further present 2 illustrative examples of how radiomic phenotyping of EAT across 2 otherwise similar EAT depots may uncover hidden phenotypes that are prognostic of downstream HF risk.

## Discussion

In this study we present a novel fully automated machine learning algorithm, FRP_HF_, that uses deep radiomic phenotyping of EAT to predict incident HF among individuals undergoing CCTA (Central Illustration). In a population of more than 70,000 individuals undergoing CCTA, FRP_HF_ outperformed models composed of traditional risk factors and demonstrated specificity for HF over competing cardiovascular outcomes. Importantly, the findings remained valid across geographically distinct datasets, key demographic subgroups, and CAD presence and severity strata. Taken together, these findings establish a new dimension to CCTA interpretation, highlighting its promising role as a screening tool for early signs of HF by uncovering clinically and prognostically relevant phenotypic information hidden in the epicardial fat depot. Integration of this automated image analysis model into clinical care may inform targeted risk modification strategies, including the precision deployment of adipose tissue-modulating therapeutics in high risk individuals.^6^

**Central Illustration:**
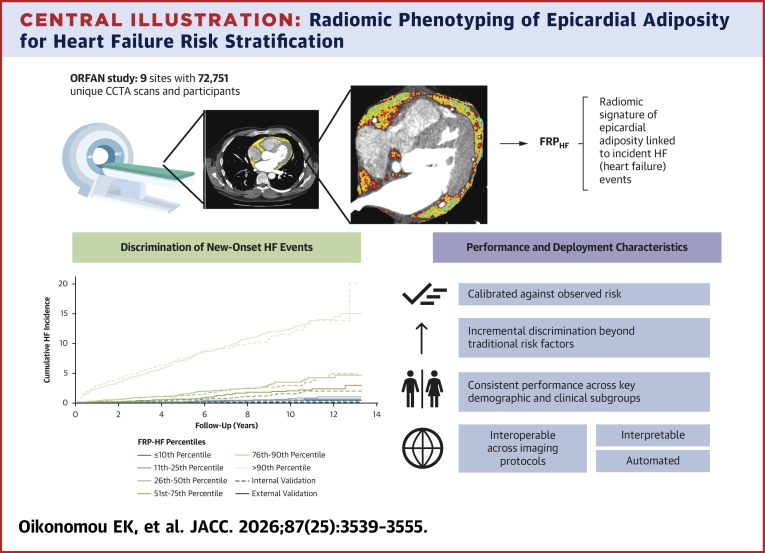
Radiomic Phenotyping of Epicardial Adiposity for Heart Failure Risk Stratification We present a radiomic fat phenotype (FRP_HF_) derived from epicardial adipose tissue on routine CCTA in the ORFAN multicenter cohort (9 sites, 72,751 scans). FRP_HF_ captures adverse adipose remodeling linked to incident HF, demonstrates graded separation of cumulative HF incidence in internal and external validation, and provides calibrated incremental discrimination beyond traditional risk factors ,with consistent performance across subgroups and imaging protocols. CCTA = coronary computed tomographic angiography; EAT = epicardial adipose tissue; FRP_HF_ = fat radiomic phenotype for heart failure; HF = heart failure.

This work is both clinically and methodologically novel. From a clinical perspective, FRP_HF_ is best interpreted as a prognostic risk-enrichment biomarker. As shown in this national cohort, FRP_HF_ consistently and reproducibly identified subgroups with disproportionately elevated long-term risk despite an otherwise low cohort-level risk of incident HF. In this context, the principal value of FRP_HF_ lies in refining risk stratification, while eventually enabling biologically grounded evaluation of preventive and myocardial-protective strategies in future prospective studies. This is further supported by evidence that EAT acts both as a sensor of myocardial inflammation and a modulator of myocardial biology, inflammation and fibrotic remodeling, through paracrine signaling molecules from and to the human heart,^9^^,^^10^^,^^32^ a bidirectional interplay that has also been described between perivascular fat and the coronary arteries.^11^^,^^33^

Our results also inform the broader epidemiologic landscape linking visceral adiposity to incident cardiovascular disease. A large number of studies have established strong associations between visceral adiposity and cardiovascular risk.^4^^,^^23^^,^^34^ Noninvasive imaging modalities, including CT,^23^^,^^34^ magnetic resonance imaging,^35^ and transthoracic echocardiography,^36^ have shown the ability to estimate EAT mass, offering accessible biomarkers of cardiometabolic risk. Efforts to derive high-risk radiomic signatures of EAT have largely relied on noncontrast CT scans (eg, as used for coronary artery calcium scoring),^34^ which have substantially lower spatial resolution than CCTA, thereby limiting the detection of submillimeter regional remodeling in EAT, which is essential to enable reliable insights into tissue biology and structure.^15^^,^^18^ Furthermore, we have shown that contrast-enhanced radiomics may detect extracellular matrix changes in the EAT, including microvascular remodeling.^15^

This study also highlights the relevance of regional disease-specific characterization of EAT across a spectrum of cardiovascular phenotypes. By evaluating the specificity of FRP_HF_ for HF events, and comparing its performance with a previously developed radiomic signature in PVAT (FRP_PVAT_),^15^ we demonstrate that distinct AT depots play divergent roles in the pathogenesis of myocardial vs vascular disease. Although FRP_PVAT_ is derived from PVAT, a subcomponent of the broader EAT depot, it is associated with ischemic but not nonischemic HF events, supporting its specificity for coronary inflammation and atherosclerosis. In contrast, FRP_HF_ is associated with both ischemic and nonischemic HF events, but not with MI, suggesting that it reflects myocardial rather than vascular biology.

There are 6 main strengths and implications arising from this work. First, we introduce the most extensively trained and validated radiomic signature of adverse EAT remodeling on CCTA and its specific links to incident HF. Second, this study represents the largest radiomic analysis of CCTA scans to date, using the largest CCTA radiomic resource linked to national EHR data, thus ensuring comprehensive capture of exposures, phenotypes, and outcomes. Third, the proposed algorithm is fully automated, eliminating the need for manual preprocessing and ensuring operator independence and scalability across imaging platforms. Fourth, our radiomic signature advances current EAT imaging standards by moving beyond conventional volumetric and attenuation features to high-dimensional radiomic descriptors that consistently offer incremental prognostic value over both traditional imaging biomarkers and clinical risk factors. Fifth, the algorithm demonstrates robust generalizability, with consistent performance across external unseen datasets, and exhibits strong specificity for HF compared with other cardiometabolic and vascular outcomes. And sixth, given the central role of CCTA in many diagnostic pathways and its emerging role in primary prevention,^37^^,^^38^ our model offers a direct path toward clinical translation. On the one hand, FRP_HF_ enables direct quantification of adverse visceral adiposity and can inform intensification of cardiometabolic risk factor optimization. On the other hand, in the context of emerging incretin-based therapies,^6^ tools such as FRP_HF_ may help broaden therapeutic indications beyond BMI-defined obesity or established comorbidities, such as type 2 diabetes mellitus, to include individuals with imaging evidence of adverse EAT remodeling. Notably, the prognostic utility of the EAT radiomic signature appears to be most pronounced in individuals without known diabetes mellitus and is preserved among those with normal BMI at baseline, further underscoring its potential to uncover latent risk not captured by conventional metrics.

### Study limitations

First, the analysis was conducted across multiple centers in the UK and may reflect local patterns of CCTA utilization that do not fully generalize to other countries. However, although CCTA has been adopted as a first-line diagnostic modality in the UK earlier than in many other health systems,^39^ in recent years its use has become increasingly standardized and more widely adopted internationally.^40^ Second, although external validation supports the generalizability of the radiomic signature, further evaluation is warranted in studies using newer scanner technologies, such as photon-counting CT systems.^41^ Third, although absolute gains in discrimination were modest, they were consistent, statistically robust, and accompanied by preserved net clinical benefit and risk reclassification, supporting the translational relevance of FRP_HF_. Fourth, regional phenotyping of distinct EAT locations or joint EAT and myocardial phenotype were not performed, because they were beyond the scope of the present work. Fifth, longitudinal changes in obesity could not be comprehensively modeled owing to incomplete follow-up anthropometric data. However, analyses of incident diabetes provided a complementary metabolic endpoint to help disentangle global adiposity burden from qualitative epicardial fat remodeling. Sixth, analyses involving hsCRP and NT-proBNP were limited to subsets of the broader cohort and should be interpreted as exploratory. Seventh, while performance was preserved across heterogeneous acquisition protocols and clinical settings, validation across additional vendor ecosystems remains an important future direction. Finally, subgroup analyses, particularly for racial subgroups with small sample sizes, should be interpreted cautiously, because sparse events can yield unstable estimates.

## Conclusions

In the largest CCTA-based radiomic analysis to date, we developed and validated an automated AI algorithm for high-throughput radiomic phenotyping of EAT, demonstrating reproducible links to incident HF. Together, these findings support EAT radiomics as a biologically informative prognostic and risk-enrichment tool to support future preventive and therapeutic studies.

### Data Availability

Individual-level imaging and clinical data are not publicly available owing to data governance and privacy restrictions, but may be made available on reasonable request to the corresponding author and principal investigator (charalambos.antoniades@cardiov.ox.ac.uk). All requests will be reviewed by the ORFAN Study Publication and Data Sharing Committee in accordance with institutional, ethical, and regulatory guidelines.

## Funding Support and Author Disclosures

This work was supported by the British Heart Foundation (CH/F/21/90009, TG/19/2/3,4831, RG/F/21/110040, and RE/24/130024 to Dr Antoniades, NHS-AI (AI_AWARD0,2013 and AI_AWARD0,2013 to Dr Antoniades), Innovate UK (104472 and 104688 to Dr Antoniades), EU Research and Innovation Action MAESTRIA (965286 to Dr Antoniades), and the NIHR Oxford Biomedical Research Centre (Cardiac and Imaging, to Drs Antoniades, Neubauer, and Channon). Dr Oikonomou received grant support via the Robert A. Winn Excellence in Clinical Trials Career Development Award (through Yale University) and the National Heart, Lung, and Blood Institute of the National Institutes of Health (F32HL1,70592) during the study. Dr Khera has received support from the National Heart, Lung, and Blood Institute of the National Institutes of Health (R01HL1,67858 and K23HL1,53775) and the National Institute on Aging of the National Institutes of Health (R01AG0,8998), outside the submitted work. Dr Oikonomou is a named co-inventor in several patents and patent applications, including 2 licensed to Caristo Diagnostics, has received royalty fees from technology licensed through the University of Oxford to Caristo Diagnostics; is a co-founder of Evidence2Health; has been a consultant for Caristo Diagnostics and Ensight-AI; and has received research support through a Robert A. Winn Excellence in Clinical Trials Career Development Award and a Wiesman Award for Excellence in Early-Career ATTR Research from Cornerstone Medical Education (through Yale University). Dr Patel, Dr Tomlins, Yogesh Sohan, Sam Fry, and Dr Siddique are employees of Caristo Diagnostics. Dr Khera is an Associate Editor of *JAMA*; receives research support, through Yale, from the Blavatnik Foundation, Bristol-Myers Squibb, Novo Nordisk, and BridgeBio; is a co-inventor in several patent application; and is a co-founder of Ensight-AI and Evidence2Health, all outside the scope of this work. Drs Shirodaria, Neubauer, Channon, and Antoniades are founders, shareholders, and nonexecutive directors of Caristo Diagnostics. Dr Antoniades owns several patents (US10695023B2, PCT/GB2017/053262, GB2018/1818049.7, GR20180100490, and GR20180100510) licensed to Caristo Diagnostics; has received honoraria from Amarin, Silence Therapeutics, Abcentra, Amgen, Nodthera, UCB, and Caristo Diagnostics; and is the immediate past chair of the British Atherosclerosis Society. All other authors have reported that they have no relationships relevant to the contents of this paper to disclose.

## References

[1] Rubino F., Cummings D.E., Eckel R.H. (2025). Definition and diagnostic criteria of clinical obesity. Lancet Diabetes Endocrinol.

[2] Lincoff A.M., Brown-Frandsen K., Colhoun H.M. (2023). Semaglutide and cardiovascular outcomes in obesity without diabetes. N Engl J Med.

[3] Oikonomou E.K., Antoniades C. (2019). The role of adipose tissue in cardiovascular health and disease. Nat Rev Cardiol.

[4] Powell-Wiley T.M., Poirier P., Burke L.E. (2021). Obesity and cardiovascular disease: a scientific statement from the American heart association. Circulation.

[5] Neeland I.J., Ross R., Després J.-P. (2019). Visceral and ectopic fat, atherosclerosis, and cardiometabolic disease: a position statement. Lancet Diabetes Endocrinol.

[6] Kusminski C.M., Perez-Tilve D., Müller T.D., DiMarchi R.D., Tschöp M.H., Scherer P.E. (2024). Transforming obesity: the advancement of multi-receptor drugs. Cell.

[7] Sattar N., Lee M.M.Y. (2025). Estimating direct tissue effects versus weight loss effects of incretin-based drugs for obesity on various chronic conditions. Lancet Diabetes Endocrinol.

[8] Iacobellis G. (2022). Epicardial adipose tissue in contemporary cardiology. Nat Rev Cardiol.

[9] Carena M.C., Badi I., Polkinghorne M. (2023). Role of human epicardial adipose tissue-derived miR-92a-3p in myocardial redox state. J Am Coll Cardiol.

[10] Antonopoulos A.S., Margaritis M., Verheule S. (2016). Mutual regulation of epicardial adipose tissue and myocardial redox state by PPAR-γ/adiponectin signalling. Circ Res.

[11] Antonopoulos A.S., Sanna F., Sabharwal N. (2017). Detecting human coronary inflammation by imaging perivascular fat. Sci Transl Med.

[12] Knuuti J., Wijns W., Saraste A. (2020). 2019 ESC guidelines for the diagnosis and management of chronic coronary syndromes. Eur Heart J.

[13] Narula J., Chandrashekhar Y., Ahmadi A. (2021). SCCT 2021 expert consensus document on coronary computed tomographic angiography: a report of the society of cardiovascular computed tomography. J Cardiovasc Comput Tomogr.

[14] Oikonomou E.K., Marwan M., Desai M.Y. (2018). Noninvasive detection of coronary inflammation using computed tomography and prediction of residual cardiovascular risk (the CRISP CT study): a post hoc analysis of prospective outcome data. Lancet.

[15] Oikonomou E.K., Williams M.C., Kotanidis C.P. (2019). A novel machine learning-derived radiotranscriptomic signature of perivascular fat improves cardiac risk prediction using coronary CT angiography. Eur Heart J.

[16] Chan K., Wahome E., Tsiachristas A. (2024). Inflammatory risk and cardiovascular events in patients without obstructive coronary artery disease: the ORFAN multicentre, longitudinal cohort study. Lancet.

[17] Berenguer R., Pastor-Juan M.D.R., Canales-Vázquez J. (2018). Radiomics of CT features may be nonreproducible and redundant: influence of CT acquisition parameters. Radiology.

[18] Kotanidis C.P., Xie C., Alexander D. (2022). Constructing custom-made radiotranscriptomic signatures of vascular inflammation from routine CT angiograms: a prospective outcomes validation study in Covid-19. Lancet Digit Health.

[19] Jastreboff A.M., Aronne L.J., Ahmad N.N. (2022). Tirzepatide once weekly for the treatment of obesity. N Engl J Med.

[20] Packer M., Zile M.R., Kramer C.M. (2025). Tirzepatide for heart failure with preserved ejection fraction and obesity. N Engl J Med.

[21] Sattar N., McGuire D.K., Pavo I. (2022). Tirzepatide cardiovascular event risk assessment: a pre-specified meta-analysis. Nat Med.

[22] Heidenreich P.A., Bozkurt B., Aguilar D. (2022). 2022 AHA/ACC/HFSA guideline for the management of heart failure: a report of the American College of Cardiology/American Heart Association Joint Committee on Clinical Practice Guidelines. J Am Coll Cardiol.

[23] West H.W., Siddique M., Williams M.C. (2023). Deep-learning for epicardial adipose tissue assessment with computed tomography: implications for cardiovascular risk prediction. JACC Cardiovasc Imaging.

[24] Fortin J.-P., Cullen N., Sheline Y.I. (2018). Harmonization of cortical thickness measurements across scanners and sites. Neuroimage.

[25] Kingma D.P., Welling M. (2019). An Introduction to Variational Autoencoders. arXiv.

[26] Cury R.C., Leipsic J., Abbara S. (2022). CAD-RADS 2.0—2022 Coronary Artery Disease-Reporting and Data System: an expert consensus document of the society of cardiovascular computed tomography (SCCT), the American College of Cardiology (ACC), the American College of Radiology (ACR), and the North America Society of Cardiovascular Imaging (NASCI). J Cardiovasc Comput Tomogr.

[27] Khan S.S., Matsushita K., Sang Y. (2024). Development and validation of the American Heart Association’s PREVENT equations. Circulation.

[28] Lundberg S., Lee S.-I. (2017). A unified approach to interpreting model predictions. arXiv.

[29] Demler O.V., Paynter N.P., Cook N.R. (2015). Tests of calibration and goodness-of-fit in the survival setting. Stat Med.

[30] Collins G.S., Moons K.G.M., Dhiman P. (2024). TRIPOD+AI statement: updated guidance for reporting clinical prediction models that use regression or machine learning methods. BMJ.

[31] Kagiyama N., Tokodi M., Hathaway Q.A. (2026). PRIME 2.0: Proposed requirements for cardiovascular imaging–related multimodal-AI evaluation: an updated checklist. JACC Cardiovasc Imaging.

[32] Packer M. (2018). Epicardial adipose tissue may mediate deleterious effects of obesity and inflammation on the myocardium. J Am Coll Cardiol.

[33] Margaritis M., Antonopoulos A.S., Digby J. (2013). Interactions between vascular wall and perivascular adipose tissue reveal novel roles for adiponectin in the regulation of endothelial nitric oxide synthase function in human vessels. Circulation.

[34] Ding J., Hsu F.-C., Harris T.B. (2009). The association of pericardial fat with incident coronary heart disease: the Multi-Ethnic Study of Atherosclerosis (MESA). Am J Clin Nutr.

[35] Kany S., Al-Alusi M.A., Rämö J.T. (2025). “Weekend warrior” physical activity and adipose tissue deposition. JACC Adv.

[36] Gustafsson B., Rovio S.P., Ruohonen S. (2024). Determinants of echocardiographic epicardial adipose tissue in a general middle-aged population—the Cardiovascular Risk in Young Finns Study. Sci Rep.

[37] Bergström G., Engström G., Björnson E. (2026). Coronary computed tomography angiography in prediction of first coronary events. JAMA.

[38] McDermott M., Meah M.N., Khaing P. (2024). Rationale and design of SCOT-HEART 2 trial: CT angiography for the prevention of myocardial infarction. JACC Cardiovasc Imaging.

[39] Moss A.J., Williams M.C., Newby D.E., Nicol E.D. (2017). The updated NICE guidelines: cardiac CT as the first-line test for coronary artery disease. Curr Cardiovasc Imaging Rep.

[40] Serruys P.W., Hara H., Garg S. (2021). Coronary computed tomographic angiography for complete assessment of coronary artery disease: JACC state of the art review. J Am Coll Cardiol.

[41] Sakai K., Shin D., Singh M. (2025). Diagnostic performance and clinical impact of photon-counting detector computed tomography in coronary artery disease. J Am Coll Cardiol.

