# Supplementary material for: Early Prediction of Heart Failure From Routine Cardiac CT Using Radiomic Phenotyping of Epicardial Fat
Source: J Am Coll Cardiol. 2026 Jun 30;87(25):3539–55. doi: 10.1016/j.jacc.2026.02.5116 (PMC13289806; doi:10.1016/j.jacc.2026.02.5116)
Supplement: Supplemental Material [file mmc1.docx]

**SUPPLEMENTAL MATERIAL**

**A. List of ORFAN Investigators**

| **First Name and Middle Initial(s)** | **Last Name** | **Institution** | **Location** |
| --- | --- | --- | --- |
| Sheena | Thomas | ORFAN core laboratory, Acute Multidisciplinary Imaging and Interventional Centre, Division of Cardiovascular Medicine, Radcliffe Department of Medicine, University of Oxford | Oxford, UK |
| Jon | Denton | ORFAN core laboratory, Acute Multidisciplinary Imaging and Interventional Centre, Division of Cardiovascular Medicine, Radcliffe Department of Medicine, University of Oxford | Oxford, UK |
| Robyn | Farrall | ORFAN core laboratory, Acute Multidisciplinary Imaging and Interventional Centre, Division of Cardiovascular Medicine, Radcliffe Department of Medicine, University of Oxford | Oxford, UK |
| Wendy | Qin | ORFAN core laboratory, Acute Multidisciplinary Imaging and Interventional Centre, Division of Cardiovascular Medicine, Radcliffe Department of Medicine, University of Oxford | Oxford, UK |
| Mary | Kasongo | ORFAN core laboratory, Acute Multidisciplinary Imaging and Interventional Centre, Division of Cardiovascular Medicine, Radcliffe Department of Medicine, University of Oxford | Oxford, UK |
| Chrisha | Ledesma | ORFAN core laboratory, Acute Multidisciplinary Imaging and Interventional Centre, Division of Cardiovascular Medicine, Radcliffe Department of Medicine, University of Oxford | Oxford, UK |
| Damaris | Darby | ORFAN core laboratory, Acute Multidisciplinary Imaging and Interventional Centre, Division of Cardiovascular Medicine, Radcliffe Department of Medicine, University of Oxford | Oxford, UK |
| Ahmad | Abdullrahman | ORFAN core laboratory, Acute Multidisciplinary Imaging and Interventional Centre, Division of Cardiovascular Medicine, Radcliffe Department of Medicine, University of Oxford | Oxford, UK |
| Bruno | Silva Santos | ORFAN core laboratory, Acute Multidisciplinary Imaging and Interventional Centre, Division of Cardiovascular Medicine, Radcliffe Department of Medicine, University of Oxford | Oxford, UK |
| Alexios S | Antonopoulos | Acute Multidisciplinary Imaging and Interventional Centre, Division of Cardiovascular Medicine, Radcliffe Department of Medicine, University of Oxford | Oxford, UK |
| Christos P | Kotanidis | Acute Multidisciplinary Imaging and Interventional Centre, Division of Cardiovascular Medicine, Radcliffe Department of Medicine, University of Oxford | Oxford, UK |
| Michail C | Mavrogiannis | ORFAN core laboratory, Acute Multidisciplinary Imaging and Interventional Centre, Division of Cardiovascular Medicine, Radcliffe Department of Medicine, University of Oxford | Oxford, UK |
| Andrew | Kelion | Oxford University Hospitals NHS Foundation Trust | Oxford, UK |
| Susan | Anthony | Oxford University Hospitals NHS Foundation Trust | Oxford, UK |
| Adrian | Banning | Oxford University Hospitals NHS Foundation Trust | Oxford, UK |
| Cheng | Xie | Division of Cardiovascular Medicine, Radcliffe Department of Medicine, University of Oxford | Oxford, UK |
| Rafail A | Kotronias | Division of Cardiovascular Medicine, Radcliffe Department of Medicine, University of Oxford | Oxford, UK |
| Lucy | Kingham | Division of Cardiovascular Medicine, Radcliffe Department of Medicine, University of Oxford | Oxford, UK |
| Rajesh K | Kharbanda | Division of Cardiovascular Medicine, Radcliffe Department of Medicine, University of Oxford | Oxford, UK |
| Chris | Mathers | Caristo Diagnostics | Oxford, UK |
| Edward | Nicol | Royal Brompton and Harefield Hospitals | London, UK |
| Tarun K | Mittal | Royal Brompton and Harefield Hospitals | London, UK |
| Jonathan | Weir-Macall | Royal Brompton and Harefield Hospitals | London, UK |
| Attila | Kardos | Milton Keynes University Hospital NHS Foundation Trust | Milton Keynes, UK |
| Anne | Rose | Milton Keynes University Hospital NHS Foundation Trust | Milton Keynes, UK |
| David | Adlam | Department of Cardiovascular Sciences and National Institute of Health Research Leicester Biomedical Research Centre, University of Leicester | Leicester, UK |
| George | Hudson | Department of Cardiovascular Sciences and National Institute of Health Research Leicester Biomedical Research Centre, University of Leicester | Leicester, UK |
| Amrita | Bajaj | Department of Cardiovascular Sciences and National Institute of Health Research Leicester Biomedical Research Centre, University of Leicester | Leicester, UK |
| Intrajeet | Das | Department of Cardiovascular Sciences and National Institute of Health Research Leicester Biomedical Research Centre, University of Leicester | Leicester, UK |
| Aparna | Deshpande | Department of Cardiovascular Sciences and National Institute of Health Research Leicester Biomedical Research Centre, University of Leicester | Leicester, UK |
| Praveen | Rao | Department of Cardiovascular Sciences and National Institute of Health Research Leicester Biomedical Research Centre, University of Leicester | Leicester, UK |
| Dan | Lawday | Department of Cardiovascular Sciences and National Institute of Health Research Leicester Biomedical Research Centre, University of Leicester | Leicester, UK |
| Francesca | Pugliese | Barts Heart Centre, St Bartholomew's Hospital, Barts Health NHS Trust; William Harvey Research Institute, Barts and The London School of Medicine and Dentistry, Queen Mary University of London | London, UK |
| Steffen E | Petersen | Barts Heart Centre, St Bartholomew's Hospital, Barts Health NHS Trust; William Harvey Research Institute, Barts and The London School of Medicine and Dentistry, Queen Mary University of London | London, UK |
| Saeed | Mirsadraee | Royal Brompton and Harefield NHS Foundation Trust | London, UK |
| Nicholas | Screaton | Royal Papworth Hospital | Cambridge, UK |
| Jonathan | Rodrigues | Royal United Hospitals Bath NHS Foundation Trust and Department of Health, University of Bath, | Bath, UK |
| David | Murphy | Royal United Hospitals Bath NHS Foundation Trust and Department of Health, University of Bath, | Bath, UK |
| Benjamin | Hudson | Royal United Hospitals Bath NHS Foundation Trust and Department of Health, University of Bath, | Bath, UK |
| John | Graby | Royal United Hospitals Bath NHS Foundation Trust and Department of Health, University of Bath, | Bath, UK |
| Colin | Berry | NHS Greater Glasgow and Clyde | Glasgow, UK |
| Mohamed | Marwan | Department of Cardiology, Friedrich-Alexander-Universität Erlangen-Nürnberg | Erlangen, Germany |
| Pál | Maurovich-Horvat | Department of Radiology, MTA-SE Cardiovascular Imaging Research Group | Budapest, Hungary |
| Guo-Wei | He | TEDA International Cardiovascular Hospital | Tianjin, China |
| Wen-Hua | Lin | TEDA International Cardiovascular Hospital | Tianjin, China |
| Li-Juan | Fan | TEDA International Cardiovascular Hospital | Tianjin, China |
| Naohiko | Takahashi | Oita University | Japan |
| Hidekazu | Kondo | Oita University | Japan |
| Neng | Dai | Fudan University | Shanghai, China |
| Junbo | Ge | Fudan University | Shanghai, China |
| Bon-Kwon | Koo | Seoul National University | Seoul, South Korea |
| Marco | Guglielmo | Centro Cardiologico Monzino IRCCS, University of Milan | Milan, Italy |
| Gianluca | Pontone | Centro Cardiologico Monzino IRCCS, University of Milan | Milan, Italy |
| Ron | Blankstein | Brigham and Women’s Hospital | Boston, USA |
| Daniel | Huck | Brigham and Women’s Hospital | Boston, USA |
| Theodora | Benedek | University of Medicine and Pharmacy of Tirgu Mures | Romania |
| Ronak | Rajani | Guy’s and St Thomas’ NHS Foundation Trust | London, UK |
| Dijana | Vilic | Guy’s and St Thomas’ NHS Foundation Trust | London, UK |
| Haleema | Aljazzaf | Guy’s and St Thomas’ NHS Foundation Trust | London, UK |
| Mak S | Mun | Guy’s and St Thomas’ NHS Foundation Trust | London, UK |
| Giulia | Benedetti | Guy’s and St Thomas’ NHS Foundation Trust | London, UK |
| Rebecca L | Preston | Guy’s and St Thomas’ NHS Foundation Trust | London, UK |
| Zahra | Raisi-Estabragh | Queen Mary University of London | London, UK |
| Derek L | Connolly | Sandwell and West Birmingham Hospitals NHS Trust | Birmingham, UK |
| Vinoda | Sharma | Sandwell and West Birmingham Hospitals NHS Trust | Birmingham, UK |
| Rebecca | Grenfell | Sandwell and West Birmingham Hospitals NHS Trust | Birmingham, UK |
| William | Bradlow | University Hospitals Birmingham (UHB) NHS Trust | Birmingham, UK |
| Matthias | Schmitt | University Hospital of Manchester Foundation Trust | Manchester, UK |
| Fabiano | Serfaty | Serfaty Clinicas, Rio de Janeiro | Brazil |
| Ilan | Gottlieb | Casa de Saúde São José, Rio de Janeiro | Brazil |
| Mario FT | Neves | Universidade do Estado do Rio de Janeiro | Rio de Janeiro, Brazil |
| David E | Newby | University of Edinburgh | Edinburgh, UK |
| Marc R | Dweck | University of Edinburgh | Edinburgh, UK |
| Bernard J | Gersh | Mayo Clinic College of Medicine | Minnesota, USA |
| Milind | Desai | Cleveland Clinic Heart and Vascular Institute, Cleveland, | OH, USA |
| Stéphane | Hatem | Foundation for Innovation in Cardiometabolism and Nutrition (ICAN) | Paris, France |
| Alban | Redheuil | Sorbonne Université, Faculté de Médecine Pierre et Marie Curie | Paris, France |
| Georgios | Benetos | Lefkos Stavros Clinic Athens | Greece |
| Meinrad | Beer | Universitätsklinikum Ulm | Germany |
| Gastón A | Rodriguez-Granillo | ENERI Medical Institute | Argentina |
| Joseph | Selvanayagam | South Australia Health and Medical Research Institute | Adelaide, Australia |
| Francisco | Lopez-Jimenez | Mayo Clinic | Rochester, USA |
| Ruben | De Bosscher | University Hospitals Leuven | Belgium |
| Alain | Tavildari | Cardiovista | France |
| Gemma | Figtree | University of Sydney | Australia |
| Ibrahim | Danad | Amsterdam University Medical Centers | Netherlands |
| Ronney | Shantouf | Cleveland Clinic Abu Dhabi | UAE |
| Bas | Kietselaer | Zuyderland Medical Centre | Heerlen, Netherlands |
| Dimitris | Tousoulis | University of Athens | Greece |
| George | Dangas | Mount Sinai School of Medicine | New York, USA |
| Nehal N | Mehta | National Institutes of Health, National Heart, Lung, and Blood Institute | Bethesda, Maryland, USA |
| Vijay | Kunadian | Newcastle Upon Tyne Hospitals NHS Foundation Trust | Newcastle, UK |
| Timothy A | Fairbairn | Liverpool Heart and Chest Hospital NHS Foundation Trust | Liverpool, UK |
| Vasvi | Singh | HCA Healthcare Kansas City Program | KS, USA |
| Stephen | Bloom | HCA Healthcare Kansas City Program | KS, USA |
| Marly | Van Assen | Emory University | Atlanta, GA |

B**. SUPPLEMENTAL METHODS**

**Technical details on scan pre-processing and segmentation**

***Pre-processing of CCTA (coronary computed tomography angiography) scans:*** First, we filtered non-CCTA scans, including computed tomography pulmonary angiograms (CTPA), head, neck, and lung scans, as well as calcium scores, using a range of DICOM (Digital Imaging and Communications in Medicine) tags. Each DICOM tag, represented as a (Group, Element) pair in hexadecimal format, defines the category and type of the data element, enabling standardized identification of scan type and content. Whenever available, we selected series acquired at approximately 75% of the cardiac cycle. When this information was unavailable, we included all CCTA series. We determined the cardiac phase (systolic or diastolic) from the series description DICOM tag. In some cases, series contained stacked data requiring separation based on scanner-specific DICOM tags. From a total of 90,046 scans, we excluded 7,388 for the following reasons: 245 scans involved patients under 18 years of age; 2 scans were severely distorted and non-analyzable; 4 scans had fewer than 150 DICOM images and were considered too small for analysis; 7 scans contained only non-CCTA or non-contrast series; and 7,130 scans had slice thicknesses greater than 1 mm or were not CCTA studies. After exclusions, we retained 82,658 analyzable scans from ORFAN (UK sites only) for further analysis.

***Development and validation of the pericardial segmentation model:*** Pericardial segmentations were performed by using a 2D Res-U-Net deep learning segmentation model embedded into the CaRi-Heart v2.5 medical device (Caristo Diagnostics, Oxford UK). The training dataset included scans from 5,700 individuals aged 19 to 101 years, with varied scan acquisition parameters designed to reflect the diversity of the ORFAN Arm 4 cohort. Specifically, we included the following parameter ranges:

- *Age:* 19 to 101 years
- *Sex:* Male and Female
- *Tube Voltage (kVp):* 70, 80, 90, 100, 120, 135, 140, 150
- *Slice Thickness:* 0.5 mm, 0.6 mm, 0.625 mm, 0.75 mm
- *Scanner Types:* SIEMENS Sensation 64; SIEMENS SOMATOM Drive 64, Definition Flash 64, Definition Edge 64, Definition AS+ 64, Definition 32, Force 96; TOSHIBA Aquilion; GE MEDICAL SYSTEMS Revolution CT 256, CT 224, CT 160, CT 128
- *Convolution Kernels:* B25f, Bv38f, 2, FC43, B26f, FC30, STANDARD, B30f, FC23, STANDARD2, FC02, B46f, FC05, FC03, I26f, 3, FC04, B35f, FC42, D30f, Bv36d, 3

We compiled the model using the Adam optimizer with a learning rate of 1×10⁻⁵, β₁ = 0.9, β₂ = 0.999, ε = 0.01, and a weight decay of 1.99×10⁻⁷. We used Jaccard Distance Loss as the loss function, chosen for its effectiveness in penalizing segmentation overlap mismatches. During training, we monitored performance using Binary Cross-Entropy, Binary Accuracy (TensorFlow metrics), and a custom Jaccard Distance Metric. Training was performed using a TensorFlow pipeline optimized for efficient batching and preprocessing of TFRecords. We updated weights iteratively via backpropagation, minimizing the loss function while evaluating the selected metrics on both training and validation sets.

We validated the segmentation model using Dice and Jaccard similarity scores, calculated against a manually validated and corrected reference dataset. On a set of 800 previously unseen ORFAN Arm 4 scans from UK and international sites, we achieved a mean Dice score of 0.95 and a Jaccard similarity score of 0.98. The Concordance Correlation Coefficient (CCC) between predicted and manually measured epicardial fat volumes was 0.99. We subsequently applied the model to the entire ORFAN Arm 4 dataset.

***Quality control of segmentations:*** We implemented a quality control (QC) process on the predicted epicardial fat segmentations across the available scans. Initially, we used a semi-automatic QC method that flagged potentially flawed segmentations based on predefined volume thresholds. We also implemented an automated system to identify outliers or suspect predictions, which were manually reviewed and corrected as needed.

We used predicted epicardial AT volume as the primary QC parameter, with observed values ranging from 0 cm³ to 2,800 cm³. Given typical heart volumes ranging from 500 cm³ to 1,700 cm³, and up to 2,000 cm³ in cases of cardiac dilation, we flagged segmentations with volumes <500 cm³ or >1,700 cm³ for manual review. A total of 2,000 segmentations required manual QC. Common issues prompting QC included: motion artifacts, low contrast or non-contrast image quality, metallic implants (e.g., stents, wires, surgical clips), slice misalignment or gaps, and inappropriate contrast timing. If no alternative series were available, scans were excluded; otherwise, we selected a superior series when present.

***Radiomic feature extraction:*** We extracted a total of 1,655 radiomic features per scan. These features included:

- Shape Features
- First-Order Statistics
- Texture Features derived from:
- Gray Level Co-occurrence Matrix (GLCM)
- Gray Level Run Length Matrix (GLRLM)
- Gray Level Size Zone Matrix (GLSZM)
- Gray Level Dependence Matrix (GLDM)
- Neighboring Gray Tone Difference Matrix (NGTDM)

We computed these features on both the original images and on filtered images. The image transformations included:

- Laplacian of Gaussian (LoG) with sigma values: 1, 2, 3, 4, 5 mm
- Wavelet Transforms: LLH, LHL, LHH, HLL, HLH, HHL
- Mathematical Transforms: Square, Square Root, Logarithm, Exponential

We used a fixed bin width of 25 for all texture features. All scans were resampled to a uniform voxel size of 0.5 × 0.5 × 0.5 mm using Lanczos interpolation, which provides high-fidelity resampling by applying a sinc function and its windowed variant to preserve image detail and minimize aliasing artifacts. For radiomic mask resampling, we applied nearest-neighbor interpolation, which maintains the integrity of discrete label values. This method prevents averaging or distortion of binary segmentation masks, although it may introduce stair-step artifacts during upscaling. To isolate epicardial AT, we thresholded voxel intensities within a Hounsfield unit (HU) range of –190 to –30. Finally, to accelerate processing, we parallelized extraction using 112 CPUs across multiple threads. This enabled completion of radiomic feature extraction for the entire dataset in approximately 10 days.

***Perivascular AT segmentation:*** Pericoronary AT was isolated from proximal segments of the three major coronary vessels (right coronary artery (RCA), left anterior descending artery (LAD), and left circumflex artery (LCX)), in line with the methods and definitions described in our previous work by using the automated segmentation pipeline of the CaRi-Heart v2.5. Like in earlier investigations,^1–3^ we used corrected computational thresholding within an attenuation range of -190 to -30 HU. For each vessel, we extracted a total of 1,655 radiomic features. This required approximately 1 to 3 minutes per case depending on the segmentation size. To accelerate processing, we employed 112 CPUs for parallelized, multi-threaded feature extraction. Radiomic analysis for each vessel took about two days, with all three vessels processed within a week.

***Calculated body mass index (BMI) at the time of CT imaging:*** To estimate BMI across the entire ORFAN cohort among individuals with missing height and weight data, we created a novel deep learning-based imputation method that leverages direct anthropometric modeling rather than statistical imputation approaches. To achieve this, we trained a modified 3D (three-dimensional) ResNet-34 model on a selected subgroup of individuals with complete measurements to jointly predict height and weight from coronary CT angiography (CCTA) scans, while also accounting for age, and sex. Our model architecture processed volumetric CT images standardized to a 256×256×256 resolution with normalized intensities. Age and sex were then included as auxiliary tabular inputs and integrated via a parallel pathway with dropout regularization. Features extracted through volumetric ResNet blocks and global average pooling were combined with tabular data and passed through fully connected layers to produce simultaneous height and weight predictions. In summary, the model was trained on 6,502 participants using MSE (mean squared error) loss and AdamW optimization, with an 80/20 train-validation split. Before deployed across the cohort including in this study, external validation on an independent subset of 3,766 participants confirmed strong correlation between observed and estimated measurements (weight: Spearman’s rho 0.79, *P*<0.001; heigh: Spearman’s rho 0.73, *P*<0.001). After validation, the model was deployed to the scans of participants with complete missing height and weight parameters.

**C. SUPPLEMENTAL TABLES**

**Supplemental Table 1. Key diagnostic and procedural code definitions used in this manuscript.**

| **Diagnosis** | **e.g., ICD-10** |
| --- | --- |
| Hypertension | I10.x, I11.x, I12.x, I13.x, I67.4, O10.x, O11.x |
| Diabetes Mellitus | E10.x, E11.x, E12.x, E13.x, E14.x, O24.0, O24.1, O24.2, O24.3, O24.9 |
| Ischemic Heart Disease (IHD) | I20.x, I21.x, I22.x, I23.x, I24.x, I25.x, Z95.1, Z95.5 |
| (Acute) Myocardial Infarction (MI) | I21.x, I22.x, I23.x, I24.0, I24.8, I24.9 |
| Chronic Kidney Disease (CKD) | I12.x, I13.x, N18.x, Z49.x |
| Cardiomyopathy | I42.0, I42.1, I42.2, I42.5, I42.8, I42.9, I43.1, I43.8 |
| Heart Failure | I11.0, I13.0, I13.2, I50.x |
| Aortic Valve Disease | I06.x I08.0 I08.2 I08.3 I39.1 I35.x |
| Mitral Valve Disease | I05.x I34.x I08.0 I08.1 I08.3 I39.0 |
| Cardiac Death (Mortality) | Cause of death defined as: I11*, I13*, I20*, I21*, I22*, I23*, I24*, I25*, I30*, I31*, I32*, I33*, I34*, I35*, I36*, I37*, I38*, I39*, I40*, I41*, I42*, I43*, I44*, I45*, I46*, I47*, I48*, I49*, I50*, I51*, I52* |
| **Procedure** | **e.g., OPCS-4** |
| Orthotopic heart transplantation | K01 (K01.x); K02 (K02.x) + clinical reports |
| Ventricular assist device implantation | K54 (K54.x) + clinical reports |
| **ICD:** International Classification of Diseases; **OPCS:** OPCS Classification of Interventions and Procedures. | |

**Supplemental Table 2 | Unadjusted and adjusted associations with incidence of new-onset heart failure (HF).**

|  | **Internal validation set**  (HR [95%CI]  per 25 percentile increments,  *P*-value) | **External validation set**  (HR [95%CI]  per 25 percentile increments, *P*-value) |
| --- | --- | --- |
| **Unadjusted model** | 5.69 [4.65–6.95], *P*<0.001 | 5.86 [4.76–7.21], *P*<0.001 |
| **Age and sex-adjusted** | 4.51 [3.63–5.60], *P*<0.001 | 4.71 [3.74–5.94], *P*<0.001 |
| **Fully adjusted model*** | 3.90 [3.13–4.84], *P*<0.001 | 3.79 [3.01–4.76], *P*<0.001 |
| **Fully adjusted model* with center-level clustering** | 3.91[3.28–4.66], *P*<0.001 | 3.85 [2.10–7.03], *P*<0.001 |
| **Competing risk model**** | 3.83 [2.94–4.99], *P*<0.001 | 3.86 [2.82–5.29], *P*<0.001 |
| *Fully adjusted model includes the following covariates: age, sex, epicardial adipose tissue volume, body mass index, CAD-RADS 2.0 classification, hypertension, diabetes mellitus, chronic kidney disease, history of ischemic heart disease, peripheral arterial disease, or stroke.  **Model with all-cause mortality included as competing risk. | | |

**D. SUPPLEMENTAL FIGURES**

**
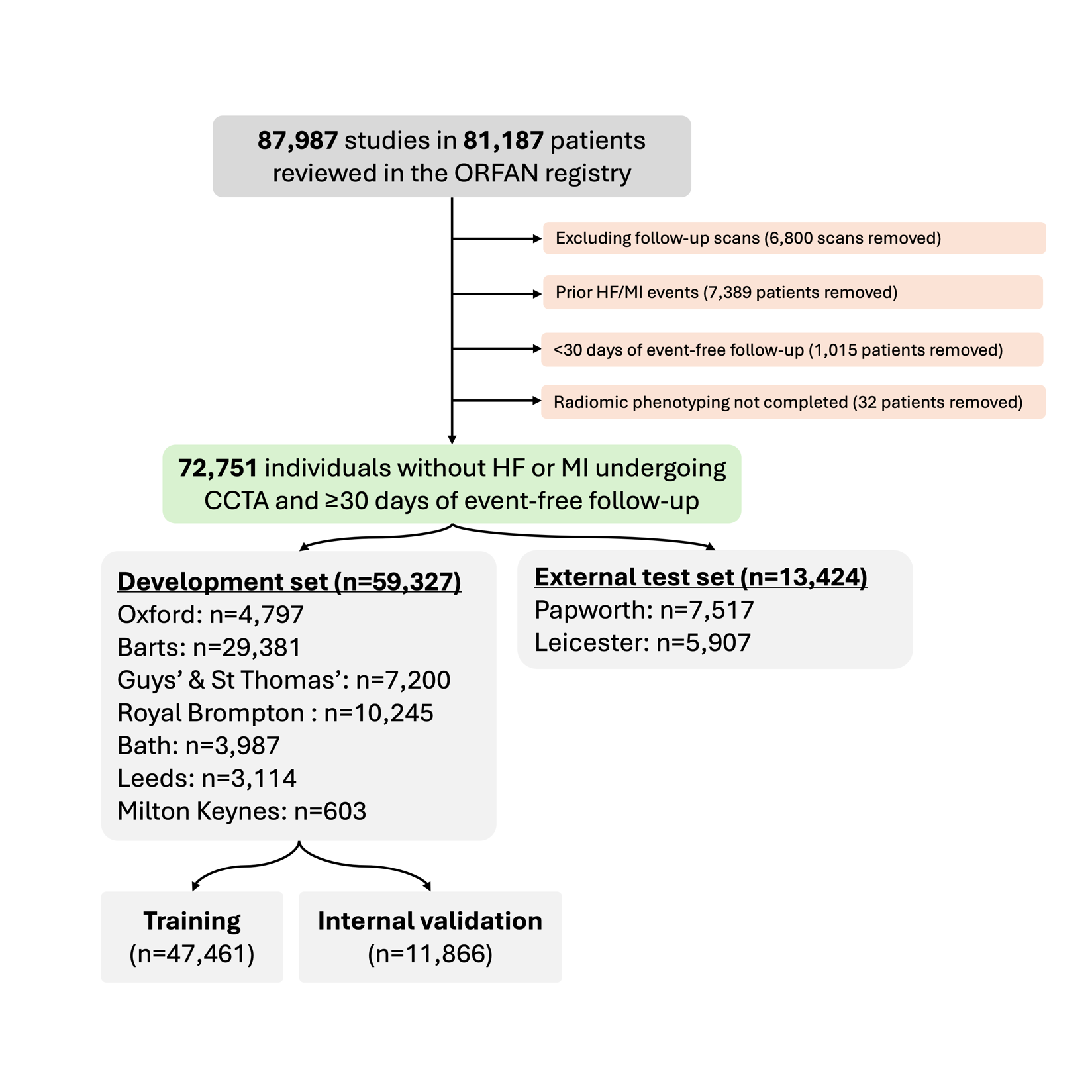
**

**Supplemental Figure 1 | Study population flowchart.** Overview of inclusions and exclusions applied to the ORFAN registry to identify the eligible population for model development and testing. *CCTA: coronary computed tomography angiography; HF: heart failure; MI: myocardial infarction; ORFAN: Oxford Risk Factors and Non-invasive Imaging cohort.*


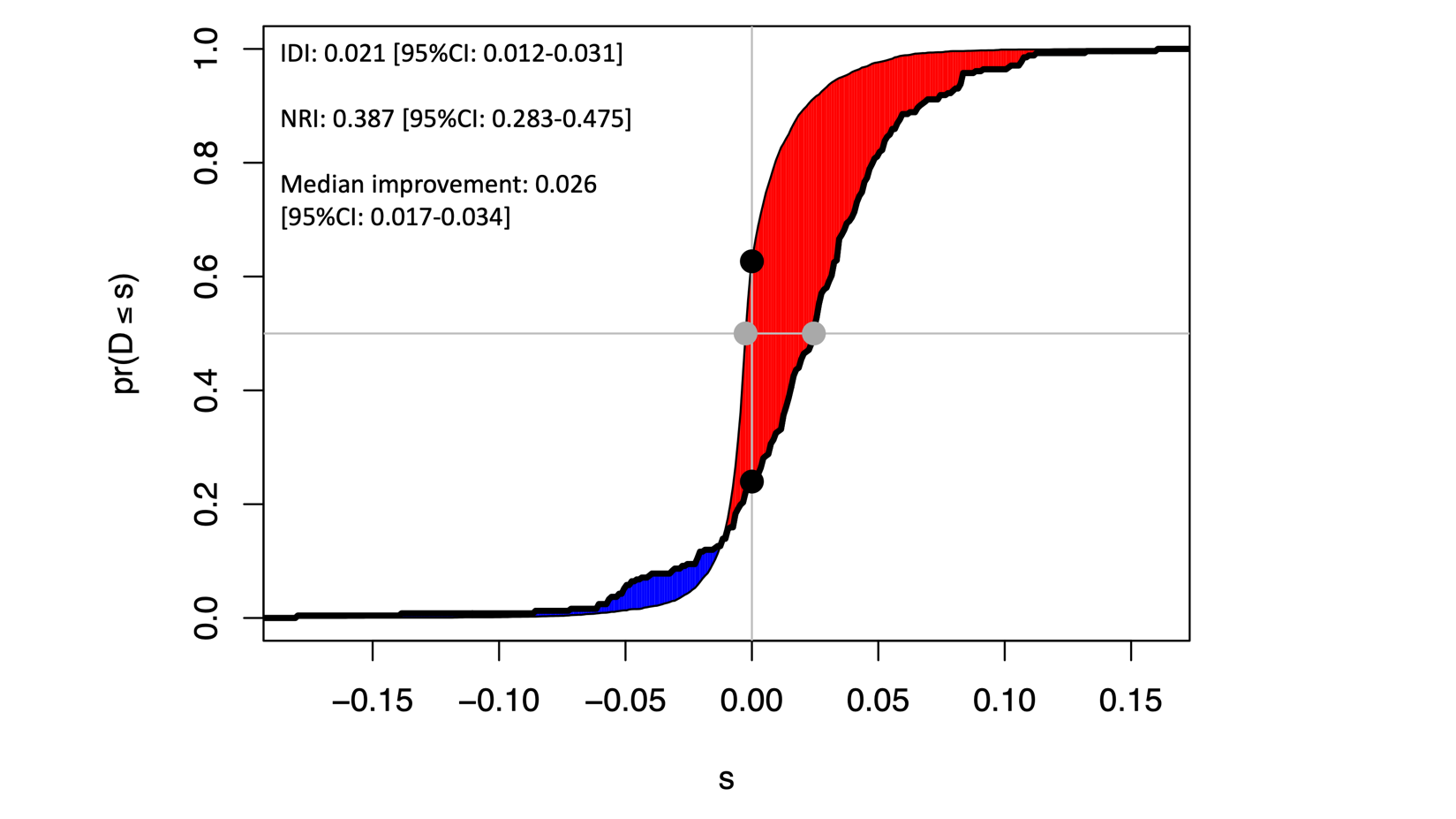


**Supplemental Figure 2. Distribution of change in predicted risk between models.** This figure shows the cumulative distribution of the change in predicted risk, denoted by s, when moving from the baseline model to the new model that incorporates FRP_HF_. The x-axis represents s (the individual-level difference in predicted probability), and the y-axis shows pr(D ≤ s), the proportion of individuals whose change in predicted risk is less than or equal to a given value of s. Values of s greater than 0 (red area) indicate that the new model assigns a higher predicted risk, while values less than 0 (blue area) indicate a lower predicted risk compared with the baseline model. The vertical line at s = 0 marks no change in risk. The horizontal line corresponds to the median of the distribution. Black points indicate the median change among individuals with and without events. The integrated discrimination improvement (IDI), net reclassification improvement (NRI), and the median improvement are shown with 95% confidence intervals, summarizing the overall improvement in risk prediction with the new model.

*
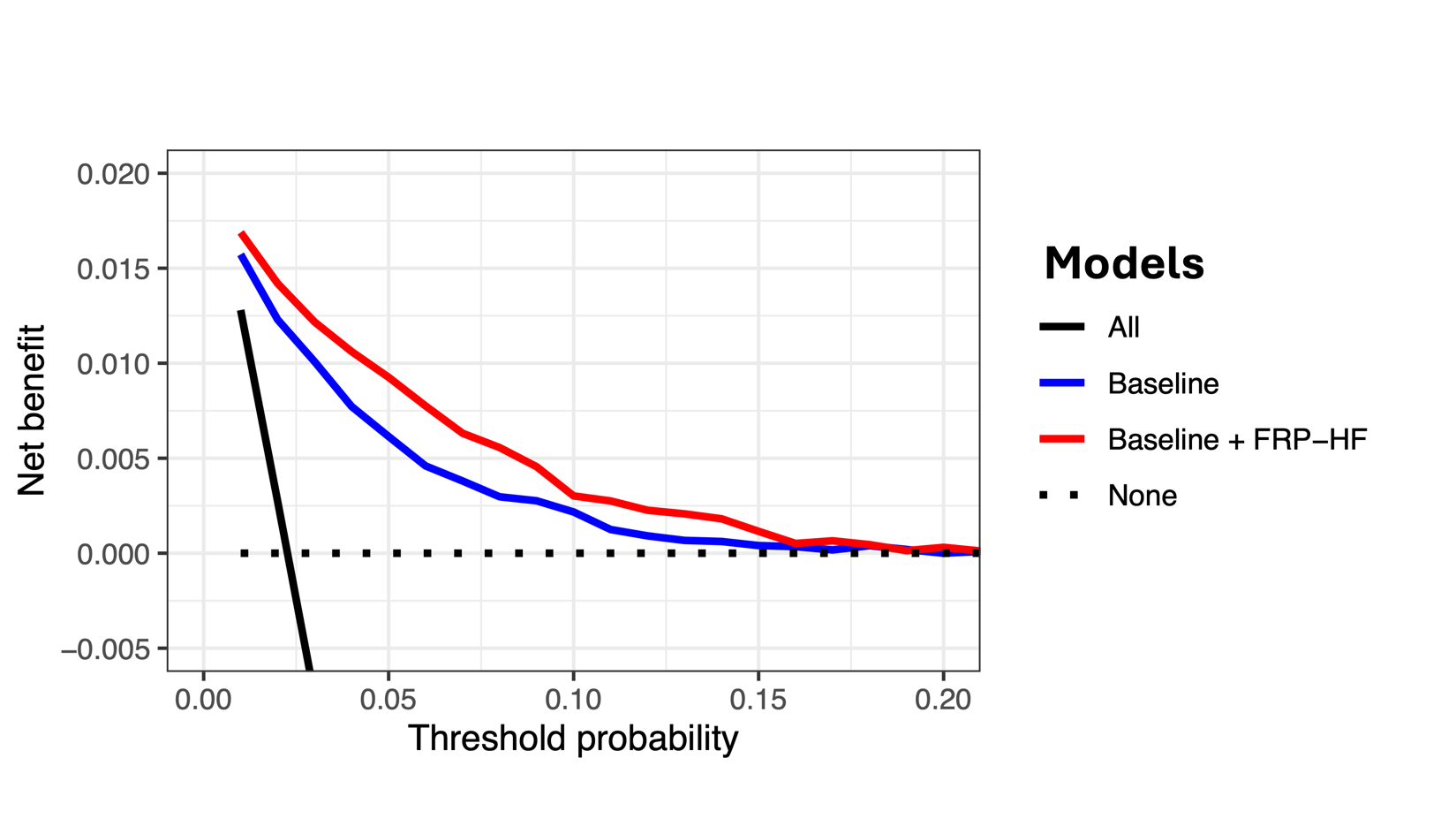
*

**Supplemental Figure 3 | Decision curve analysis for prediction of incident heart failure.** Decision curve analysis comparing the net benefit of models for 5-year incident heart failure risk across a range of threshold probabilities. The baseline model (blue line) includes age, sex, traditional cardiovascular risk factors (BMI, EAT volume, diabetes mellitus, hypertension, history of ischemic heart disease, myocardial infarction, stroke, peripheral arterial disease, chronic kidney disease) and CAD-RADS categories, whereas the blue line denotes the model that further incorporates FRP_HF_. The horizontal reference line (dotted) represents the strategy of treating no patients, whereas the continuous black line represents the strategy of treating all patients. Across clinically relevant 5-year risk thresholds (approximately 5%-15%), the model that incorporates FRP_HF_ in addition to all baseline covariates demonstrates consistently greater net benefit compared with the baseline model, supporting improved clinical utility. *BMI: body mass index;* *CAD-RADS: coronary artery disease-reporting and data system;* *EAT: epicardial adipose tissue; FRP_HF_: fat radiomic profile for heart failure.*

**
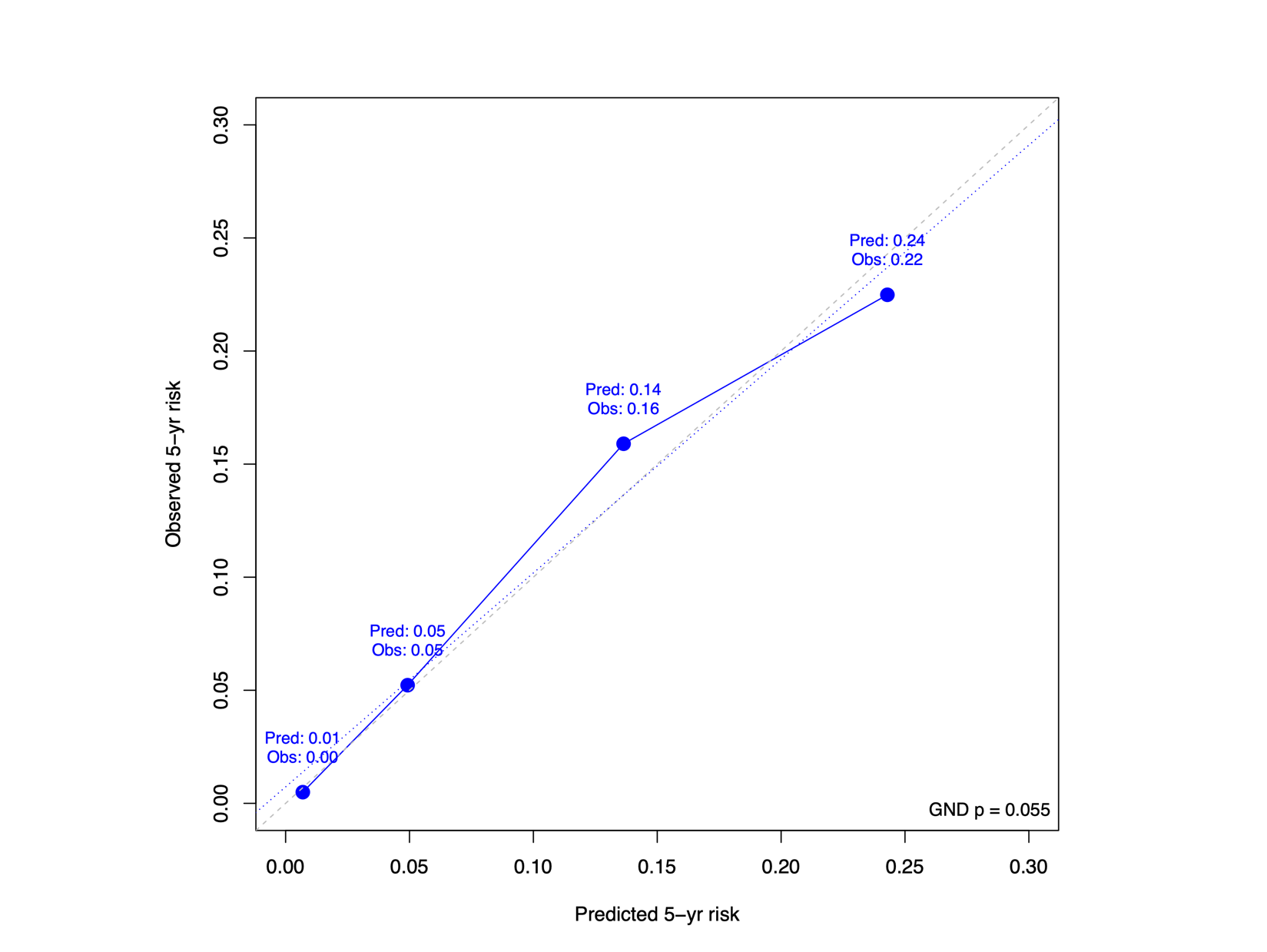
**

**Supplemental Figure 4 | Calibration of FRP_HF_ signature in the external validation set.** Observed vs predicted risk for HF-free survival at 5 years in the external validation set for a risk prediction model incorporating FRP_HF_ alongside age, sex, and traditional cardiovascular risk factors. The error bars denote 95% confidence intervals. *FRP_HF_: fat radiomic profile for heart failure risk prediction.*


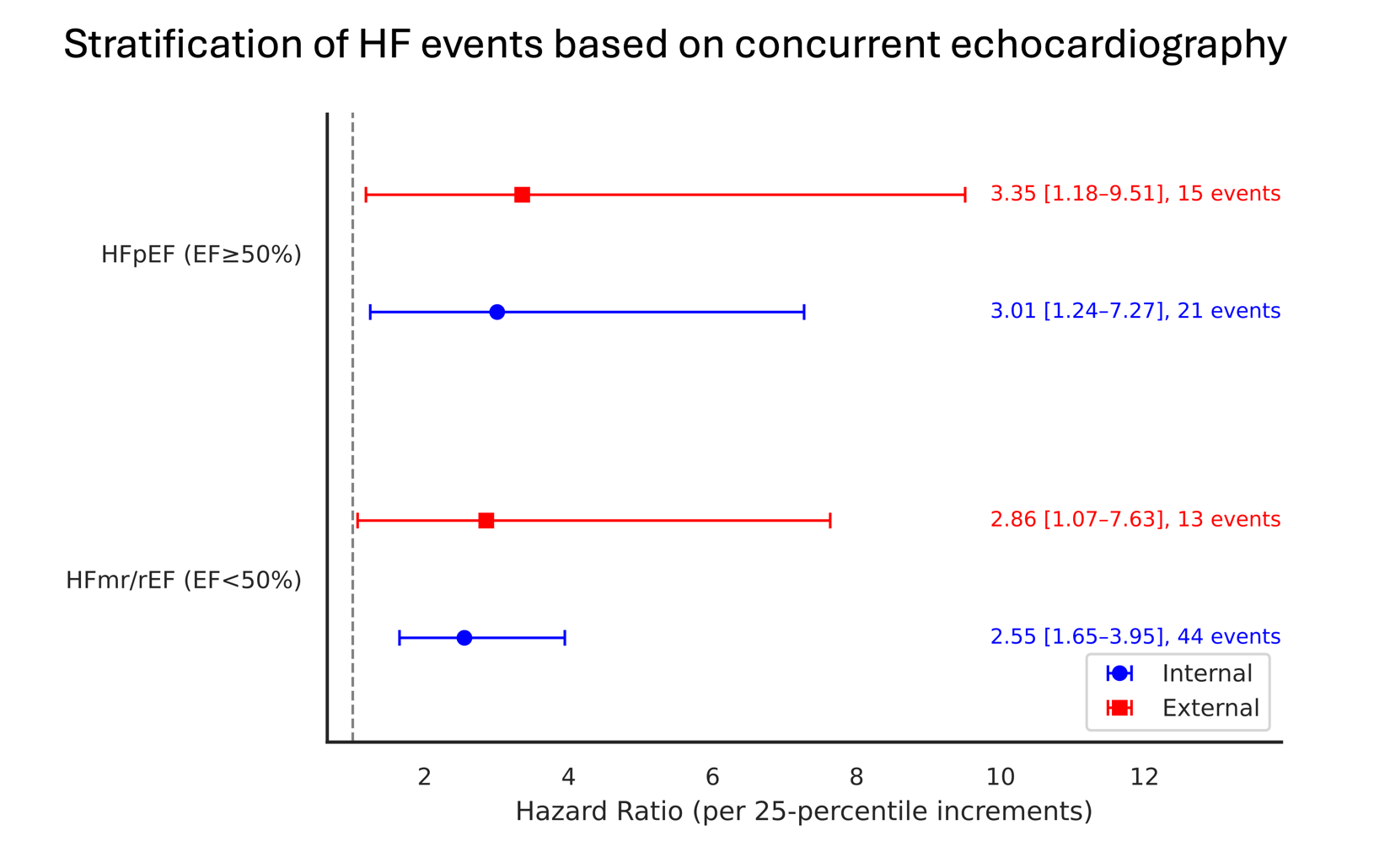


**Supplemental Figure 5 | Stratification of HF events based on left ventricular ejection fraction.** Forest plot showing the adjusted hazard ratios for the association between FRP_HF_ (per 25-percentile increments) and incident HF events, stratified by left ventricular ejection fraction (LVEF) category by transthoracic echocardiography. Results are displayed separately for the internal (blue) and external (red) validation cohorts. Error bars denote 95% confidence intervals, with the corresponding number of events indicated. *FRP_HF_: fat radiomic profile for heart failure risk prediction; HF: heart failure; HFpEF: HF with preserved EF (≥50%); HFmr/rEF: HF with mildly reduced or reduced EF (41-49%, or ≤40%, respectively); HR: hazard ratio; CI: confidence interval.*

*
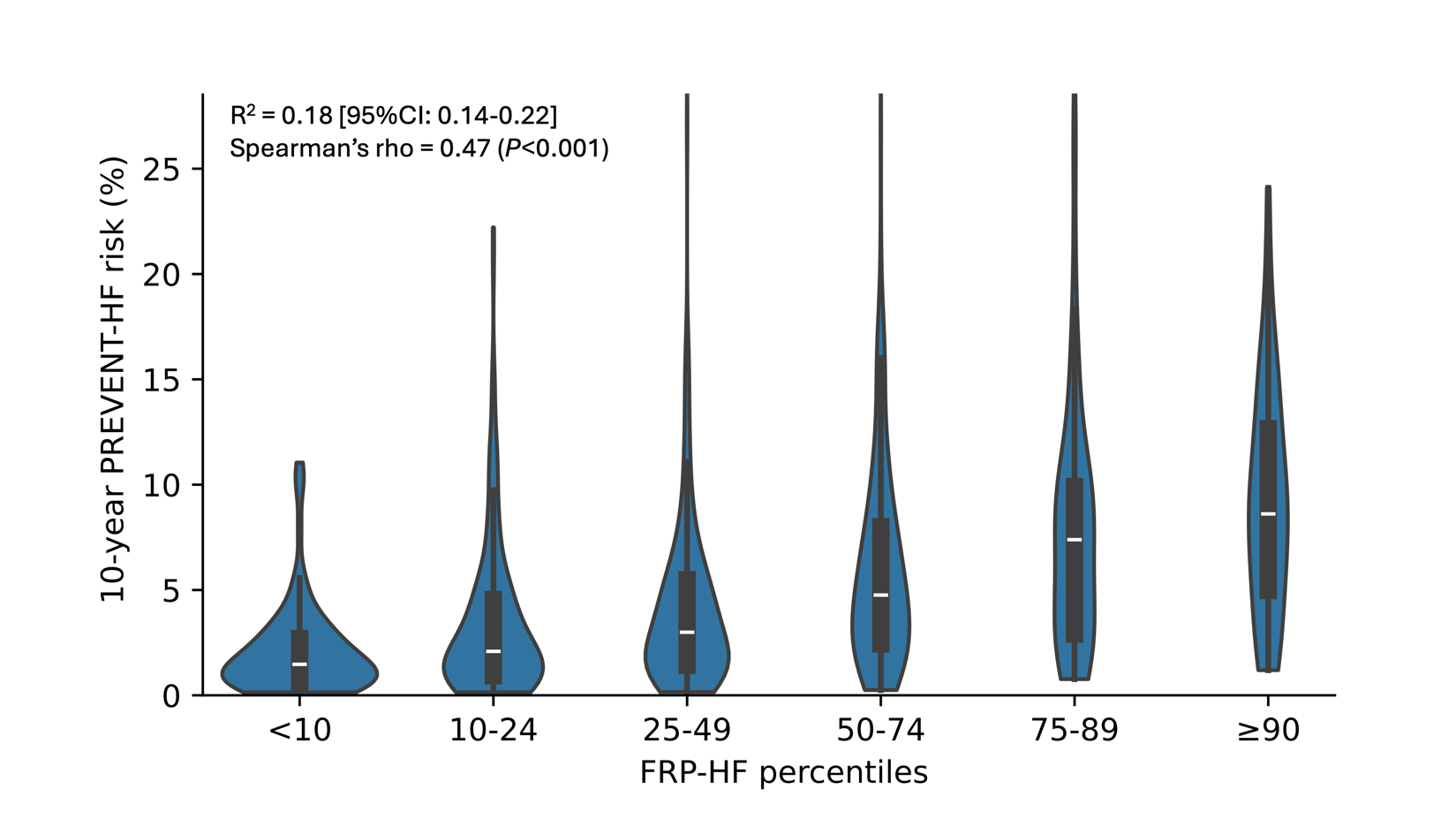
*

**Supplemental Figure 6 | Distribution of 10-year PREVENT-HF risk across FRP_HF_ percentiles.** Violin plots displaying the distribution of predicted 10-year PREVENT-HF risk (%) across categories of the FRP_HF_ score (<10^th^, 10-24^th^, 25-49^th^, 50-74^th^, 75-89^th^, and ≥90^th^ percentiles). Inner boxplots denote the interquartile range with median values; widths reflect kernel density. A graded increase in PREVENT-HF risk is observed across increasing FRP_HF_ strata. Overall correlation between FRP_HF_ and PREVENT-HF risk was moderate (R² = 0.18 [95%CI: 0.14-0.22]; total n = 1,077 from a pooled analysis of the internal and external validation sets. *FRP_HF_: fat radiomic profile for heart failure risk prediction.*

**
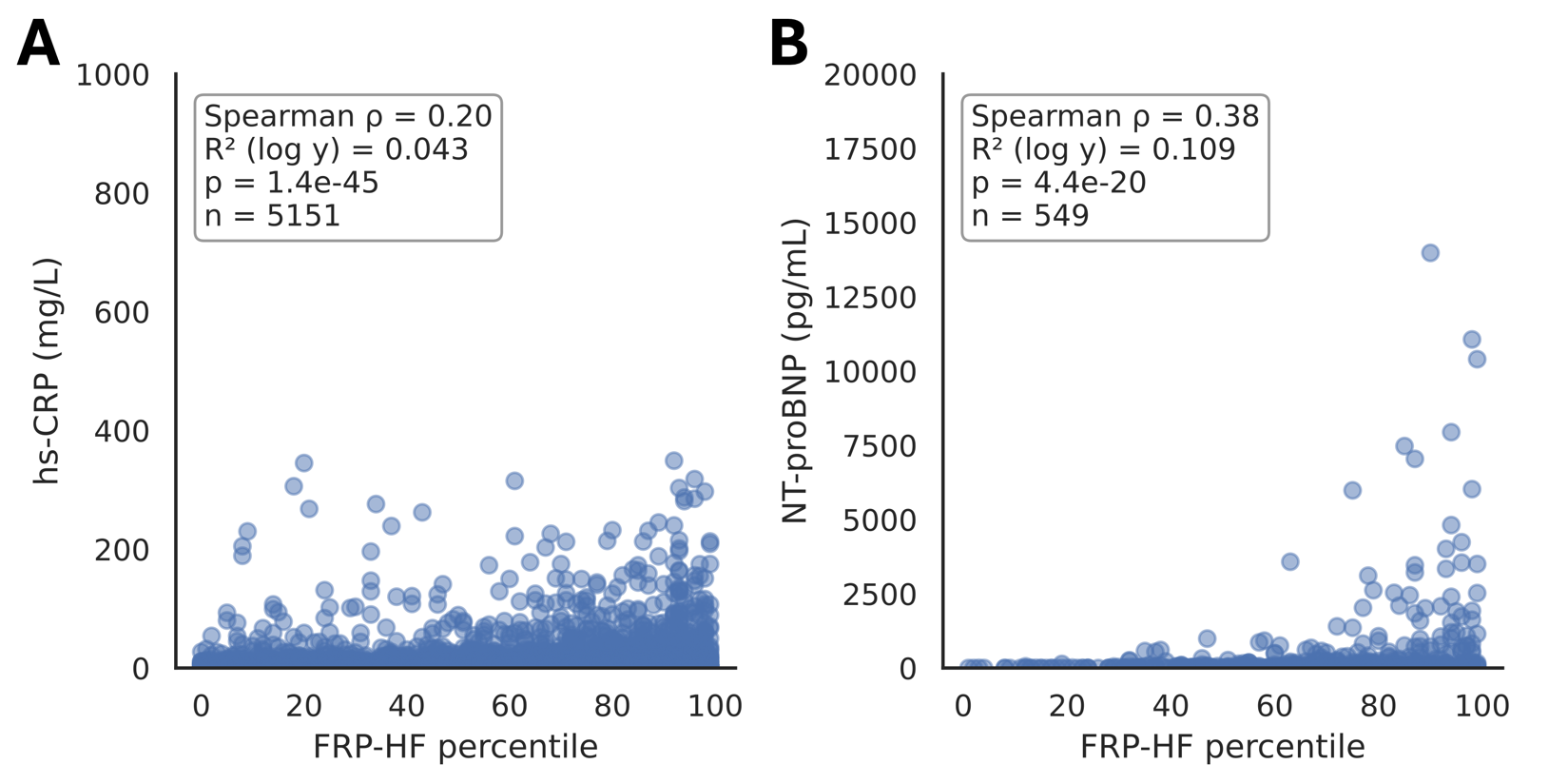
**

**Supplemental Figure 7 | Correlation between FRP_HF_ and high-sensitivity C-reactive protein (hsCRP) and N-terminal-pro-brain natriuretic peptide (NT-proBNP) levels.** Correlation (scatter) plots summarizing the distribution of **(A)** hsCRP, and **(B)** NT-proBNP across FRP_HF_ percentiles in a pooled set of participants from the internal and external validation sets who had available biomarker measurements within 1 year (before or after) the index CCTA study (n=5,151 and 549 respectively). We also present the Spearman’s rho correlation coefficient, and the R^2^ derived from a linear regression model with the log-transformed biomarker value (hsCRP or NT-proBNP) as the dependent variable, and FRP_HF_ percentile as the independent variable. *CCTA: coronary computed tomography angiography.*

**
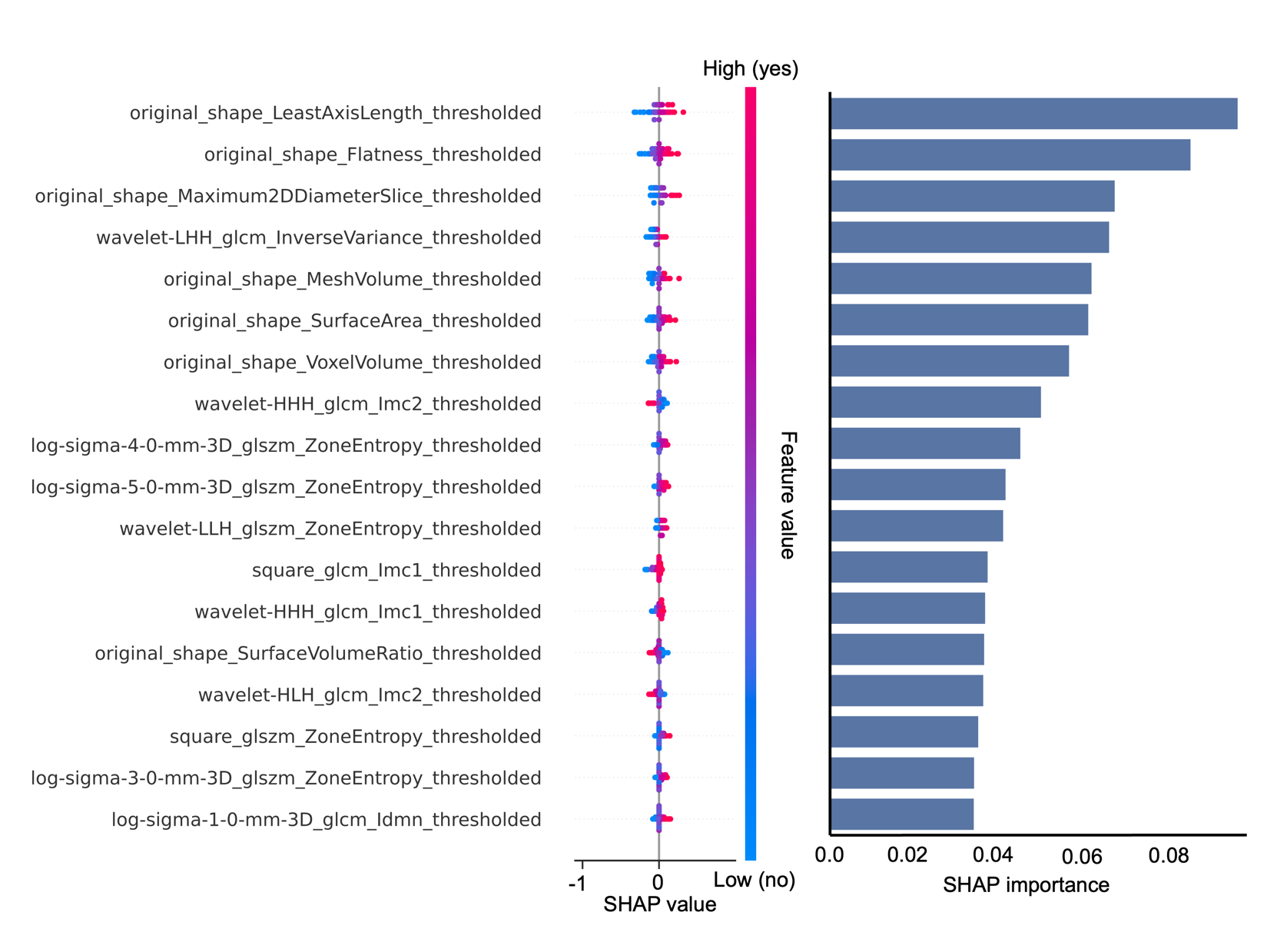
**

**Supplemental Figure 8 | Explainability analysis by SHAP.** Shapley Additive Explanation (SHAP) values for individual radiomic features for discriminating incident HF (adjusted for age and sex). The SHAP importance reflects the relative importance of individual features towards the final prediction, which ultimately incorporates all eligible features. Both geometric (shape and volume-related) as well as radiomic texture-linked features are found in the top features composing the FRP_HF_. *EAT: epicardial adipose tissue; SHAP: Shapley Additive Explanation.*

**E. SUPPLEMENTAL REFERENCES**

1. Oikonomou EK, Marwan M, Desai MY, et al. Non-invasive detection of coronary inflammation using computed tomography and prediction of residual cardiovascular risk (the CRISP CT study): a post-hoc analysis of prospective outcome data. *Lancet*. 2018;392:929–939.

2. Oikonomou EK, Williams MC, Kotanidis CP, et al. A novel machine learning-derived radiotranscriptomic signature of perivascular fat improves cardiac risk prediction using coronary CT angiography. *Eur Heart J*. 2019;40:3529–3543.

3. Chan K, Wahome E, Tsiachristas A, et al. Inflammatory risk and cardiovascular events in patients without obstructive coronary artery disease: the ORFAN multicentre, longitudinal cohort study. *Lancet*. 2024;403:2606–2618.
